# An axonal brake on striatal dopamine output by cholinergic interneurons

**DOI:** 10.1038/s41593-025-01906-5

**Published:** 2025-03-13

**Authors:** Yan-Feng Zhang, Pengwei Luan, Qinbo Qiao, Yiran He, Peter Zatka-Haas, Guofeng Zhang, Michael Z. Lin, Armin Lak, Miao Jing, Edward O. Mann, Stephanie J. Cragg

**Affiliations:** 1 Department of Physiology, Anatomy and Genetics, University of Oxford, Oxford, UK; 2 Aligning Science Across Parkinson’s Collaborative Research Network, Chevy Chase, MD USA; 3 Oxford Parkinson’s Disease Centre, University of Oxford, Oxford, UK; 4 Chinese Institute for Brain Research, Beijing, China; 5 Department of Neurosurgery, The First Affiliated Hospital of Sun Yat-sen University, Guangzhou, China; 6 Department of Neurobiology, Stanford University, Stanford, CA USA; 7 Department of Bioengineering, Stanford University, Stanford, CA USA; 8 Present Address: Department of Clinical and Biomedical Sciences, University of Exeter, Exeter, UK

**Keywords:** Neural circuits, Excitability

## Abstract

Depolarization of axons is necessary for somatic action potentials to trigger axonal neurotransmitter release. Here we show that striatal cholinergic interneurons (ChIs) and nicotinic receptors (nAChRs) on mouse dopamine axons interrupt this relationship. After nAChR-mediated depolarization, dopamine release by subsequent depolarization events was suppressed for ~100 ms. This suppression was not due to depletion of dopamine or acetylcholine, but to a limited reactivation of dopamine axons after nAChR-mediated depolarization, and is more prominent in dorsal than in ventral striatum. In vivo, nAChRs predominantly depressed dopamine release, as nAChR antagonism in dorsal striatum elevated dopamine detected with optic-fiber photometry of dopamine sensor GRAB_DA2m_ and promoted conditioned place preference. Our findings reveal that ChIs acting via nAChRs transiently limit the reactivation of dopamine axons for subsequent action potentials in dopamine neurons and therefore generate a dynamic inverse scaling of dopamine release according to ChI activity.

## Abstract (web summary)

Cholinergic interneurons act at nicotinic receptors to depress dopamine release, interrupting its relationship to dopamine neuron firing and supporting an inverse scaling of dopamine release according to cholinergic activity.

## Main

Neuronal axons propagate action potentials and, at vesicular release sites, transform these electrical impulses into neurotransmitter release to modulate target cells. Our current understanding of the physiological functions of the neurotransmitter dopamine (DA), such as the encoding of reward prediction error, has been developed substantially from recordings of action potential firing in the soma of DA neurons^1^. However, mechanisms and inputs acting selectively in or on DA axons are positioned to gate DA release^2–5^, with some in vivo evidence that striatal DA release is dissociated from DA somatic activity in some circumstances^6^. Tonically active cholinergic interneurons (ChIs) within the striatum constitute only ~2% of striatal neurons, but arborize densely, and through axo-axonic actions on DA axons can drive short-latency or ‘instantaneous’ DA release events via the activation of β2-containing-nAChRs^4,5^ and ectopic axonal generation of action potentials^7^. However, little in the way of supporting evidence has been gained in recent studies in other paradigms for the likelihood that ChIs drive or change the timing of DA release in vivo^8–10^. Conversely, ex vivo experiments have suggested that, when ChIs and DA axons are concurrently stimulated, the DA release level might be less than for the activation of DA axons alone^2^, suggesting that axonic inputs from ChIs might paradoxically limit DA release. The ability of ChIs to trigger ectopic action potentials in DA axons might sit within an alternative context of axo-axonic signal integration. Here, we test the hypothesis that activation of nAChRs can limit the amplitude of DA release, by impairing the translation of subsequent action potentials in DA axons into DA release. We find that discrete activation of ChIs and nAChRs and depolarization of DA axons can profoundly and dynamically limit the ensuing depolarization of DA axons by incoming activity for up to ~100 ms. This suppression can also occur for low levels of ChI activation that are not sufficiently strong to trigger detectable DA release. DA release in vivo is then enhanced after antagonism of nAChRs, indicating that a dominant physiological outcome of ChI activity in vivo on DA signaling in the intact brain is to operate a dynamically scaling suppression of the amplitude of DA release.

## Results

### Activation of ChIs attenuates subsequent DA release

**Fig. 1:**
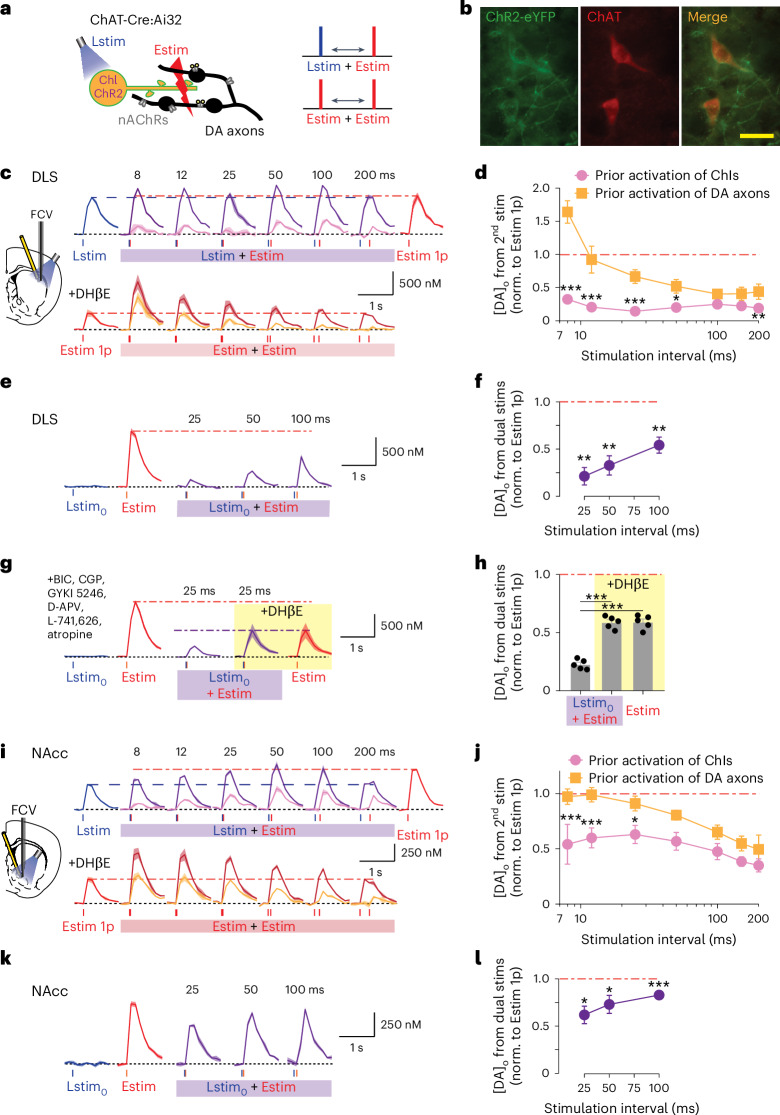
Optogenetic activation of ChIs suppresses subsequent DA release evoked by electrical stimulation. **a**, Schematic of stimulation configuration for **c**–**j**. Blue light stimulation (Lstim) of ChR2-eYFP-expressing ChIs, local electrical stimulation (Estim) in striatal slices from ChAT-Cre:Ai32 mice. **b**, ChR2-eYP expression in ChAT-immunoreactive striatal neurons. Scale bar, 40 µm. **c**,**i**, Top: mean transients from representative experiments of [DA]_o_ (± s.e.m.) evoked by a single pulse of Lstim (blue lines), or Estim (red lines), or the composite response to paired Lstim plus Estim pulses (purple) at interstimulus intervals (ISIs) of 8–200 ms in DLS (**c**) and NAcc (**i**). The pink trace shows the [DA]_o_ attributable to the paired Estim after subtraction of [DA]_o_ due to Lstim. Bottom: mean transients of [DA]_o_ (± s.e.m.) evoked by single or paired electrical pulses (red lines) in the presence of DHβE (1 µM) in DLS (**c**) and NAcc (**i**). The orange trace shows the [DA]_o_ attributable to the paired Estim after subtraction of [DA]_o_ due to single Estim. **d**,**j**, The mean peak [DA]_o_ (± s.e.m.) evoked by the paired Estim normalized (norm.) to [DA]_o_ evoked by a single Estim, versus ISI in DLS (**d**, *N* = 5 animals) and NAcc (**j**, *N* = 5 animals). *P < 0.05, **P < 0.01, ***P < 0.001, two-way analysis of variance (ANOVA) with Fisher’s least significant difference (LSD) test post hoc. **e**,**g**,**k**, Mean transients from representative experiments of [DA]_o_ (± s.e.m.) evoked by a subthreshold light pulse (Lstim_0_, blue), single Estim (red) or paired Lstim and Estim (purple) at ISIs of 25–100 ms in DLS (**e**,**g**) or NAcc (**k**). **f**,**h**,**l**, The mean peak [DA]_o_ (± s.e.m.) evoked by the dual stimuli normalized to [DA]_o_ evoked by a single Estim, versus ISI in DLS (**f**,**h**, *N* = 5 animals) and NAcc (**l**, *N* = 5 animals), in the presence of receptors antagonists for GABA_A_, GABA_B_, AMPA, NMDA, D2 and mAChRs before and after nAChRs were blocked (**g**,**h**). **P < 0.01, ***P < 0.001, two-sided one-sample *t*-test (**f**,**l**), or two-sided *t*-test versus single electrical stimulation (**h**). Horizontal dashed lines indicate peak [DA]_o_ evoked by a single stimulus of same color data as a reference. Source data

We first tested the impact of targeted activation of ChIs on subsequent DA release evoked by electrical stimulation. In ex vivo striatal slices from choline acetyltransferase (ChAT)-Cre:Ai32 mice (Fig. 1a,b), we used a blue light stimulus (Lstim) to optogenetically activate ChR2-expressing ChIs to activate nAChRs and drive instantaneous DA release (ChI-driven DA release, DA_ChI_) as previously^4^ followed 8–200 ms later by a second stimulation of a pulse of electrical stimulus (Estim), which provides a composite stimulus that drives DA release both directly via activation of DA axons (DA_DA_) and indirectly via activation of ChIs (DA_ChI_, which follows DA_DA_ with short latency, ~10 ms (refs. ^11,12^)). By measuring extracellular DA concentration ([DA]_o_) with fast-scan cyclic voltammetry (FCV), we found in dorsolateral striatum (DLS) (Fig. 1c,d) and nucleus accumbens core (NAcc) (Fig. 1i,j) that optogenetic stimulation of ChIs evoked instantaneous DA release and then depressed DA release evoked subsequently 8–200 ms later, to ~15–50% of the [DA]_o_ that could be evoked by an electrical stimulus alone. For intervals ≤50 ms in DLS and ≤25 ms in NAcc, [DA]_o_ evoked by the second stimulus was significantly less after optogenetic ChI stimulation (when normalized to initial release) than after an initial electrical stimulation in the presence of a β2-nAChR antagonist (DHβE, 1 µM) (Fig. 1c,d,i,j). These data suggest that activation of nAChRs can attenuate subsequent DA release over a short interval of up to 100 ms. Note that, with nAChRs antagonized, there is a strong inverse relationship between [DA]_o_ and interpulse interval for DA axon stimulation, as reported previously^2,13^, whereby this intrinsic short-term depression of DA release is sustained over an extended period of several seconds^2,13^.

**Extended Data Fig. 1:**
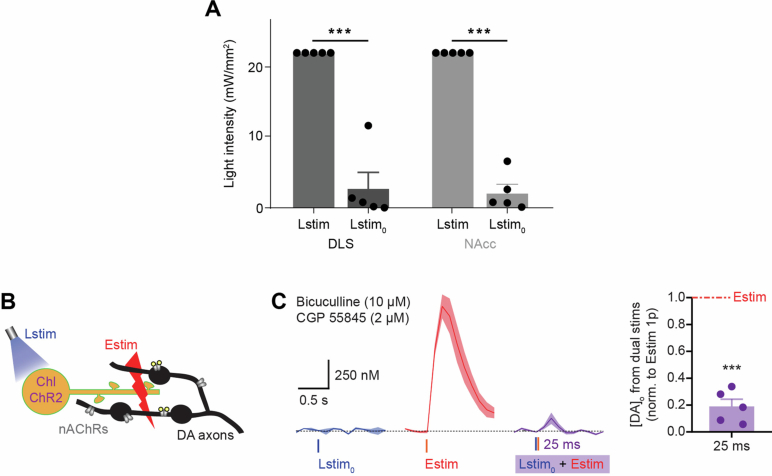
Light stimulation (Lstim_0_) intensities that are sub-threshold for evoking detectable DA release. **a**, The intensities of Lstim_0_ used to stimulate ChIs in ChAT-Cre:Ai32 mice in Fig. 1 ( ± SEM) were significantly lower than Lstim in both DLS (black) and NAcc (grey). N = 5 animals. ***P < 0.001, two-sided one-sample t-test. **b**, Schematic of stimulation configuration for data in c. Blue light stimulation (Lstim) of ChR2-eYFP-expressing ChIs, subsequent local electrical stimulation (Estim) in striatal slices from ChAT-Cre:Ai32 mice. **c**, Mean transients ( ± SEM) from representative experiments in DLS showing [DA]_o_ evoked by either a subthreshold light pulse (Lstim_0_, blue), a single Estim (red), or paired Lstim and Estim (purple) at an ISI of 25 ms when GABA_A_ and GABA_B_ receptors were inhibited using bicuculline (10 µM) and CGP 55845 hydrochloride (2 µM) respectively. Mean peak [DA]_o_ ( ± SEM) evoked by the dual stimuli normalised to [DA]_o_ evoked by a single Estim (n = 5 recordings, N = 3 animals) when GABA receptors are inhibited. *** P = 0.0001, Two-sided one sample t-test versus Estim. Source data

We explored whether the greater depression of subsequent DA release seen after optogenetic ChI activation than after electrical stimulation of DA axons in the presence of an nAChR antagonist was due to depletion of the DA vesicle pool^11^. A low intensity of light (Lstim_0_) (Extended Data Fig. 1) was applied to stimulate ChIs at a minimal level for which no instantaneous [DA]_o_ could be detected by FCV (equivalent to <0.5% of DA_ChI_ evoked by normal Lstim) (Fig. 1e,k). This Lstim_0_ stimulus ensures that >99.5% of the DA release pool is still available for release. However, we found that, even using Lstim_0_ to stimulate ChIs, the [DA]_o_ evoked by a subsequent electrical pulse was depressed to as little as 20% in DLS and 60% in NAcc of the [DA]_o_ evoked by an electrical pulse alone (Fig. 1e,f,k,l). The depression of DA release after prior ChI activation cannot then be explained solely by DA depletion, indicating that activation of ChIs or nAChRs limits subsequent DA release through a mechanism independent of DA vesicle pool availability.

We tested whether the depression of DA release was due to the initial Lstim_0_ of ChIs causing depletion of acetylcholine (ACh) and so compromising the ACh available to drive DA_ChI_ at the subsequent Estim (which is made up of DA_DA_ and DA_ChI_). We prevented DA_ChI_ altogether, by using the nAChR antagonist DHβE, and repeated the experiment to test two competing hypotheses. If the low [DA]_o_ evoked by Estim (DA_DA_ + DA_ChI_) after Lstim_0_ was due to a loss of DA_ChI_, then the [DA]_o_ evoked by Estim when nAChRs are antagonized (DA_DA_) might reach a similar but never a greater value. Conversely, if Lstim_0_ of ChIs reduces subsequent DA release evoked by Estim (DA_DA_ + DA_ChI_), then [DA]_o_ evoked by Estim when nAChRs are antagonized (DA_DA_) might be able to exceed this level of release. Testing these hypotheses in DLS, we found that the DA_DA_ evoked by Estim after Lstim_0_ was indeed significantly higher when nAChRs were antagonized than not (Fig. 1g,h) (and was equivalent to [DA]_o_ evoked by Estim when nAChRs were antagonized in the absence of prior Lstim_0_), indicating that subthreshold stimulation of ChIs actively attenuates subsequent DA release, including DA_DA_. These experiments were conducted in the presence of a cocktail of antagonists for GABA_A_ and GABA_B_ receptors (10 μM bicuculline (BIC) and 4 μM CGP 55845), α-amino-3-hydroxy-5-methyl-4-isoxazolepropionic acid (AMPA) and *N*-methyl-d-aspartate (NMDA) glutamate receptors (10 µM GYKI 5246 and 50 µM D-APV), D2 receptors (1 µM L-741,626) and mAChRs (2 µM atropine) (Fig. 1g,h), therefore allowing us to exclude the possibility that ChI-dependent attenuation of DA release was mediated by indirect activation of these other candidate receptors and transmitter networks.

**Fig. 2:**
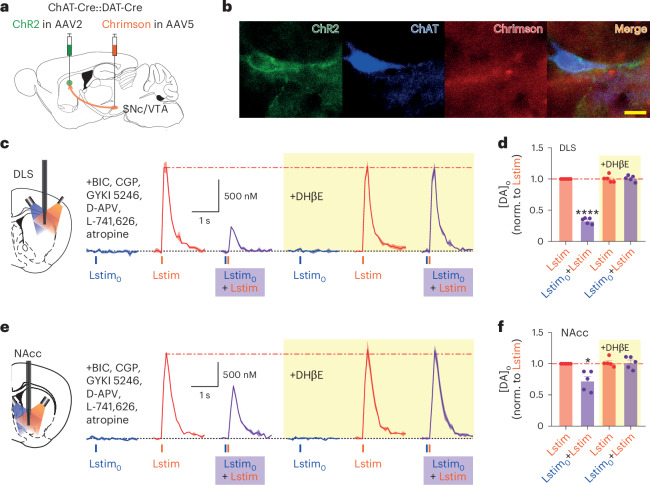
A dual optogenetic approach shows that subthreshold activation of ChIs suppresses subsequent DA release. **a**, Schematic of virus injection in double-transgenic ChAT-Cre:DAT-Cre mice. **b**, Images of a ChI with expression of ChR2 (green) colabeled with ChAT immunoreactivity (blue), alongside Chrimson positive (red) DA axons (scale bar, 20 µm). **c**,**e**, Subthreshold blue light (Lstim_0_) stimulation to preferentially activate ChR2-eYFP-expressing ChIs and orange light stimulation to preferentially activate Chrimson-expressing DA axons to drive DA release, in striatal slices in DLS (**c**) and NAcc (**e**). Mean transients of [DA]_o_ (± s.e.m.) from representative experiments evoked by a single pulse of blue Lstim_0_ (blue lines), or orange Lstim (orange lines), or the response to paired blue Lstim_0_ plus orange Lstim (purple shading) at an interval of 25 ms, in the presence of receptors antagonists for GABA_A_, GABA_B_, AMPA, NMDA, D_2_ and mAChRs. The yellow shading indicates the presence of DHβE (1 µM). **d**,**f**, The mean peak [DA]_o_ (± s.e.m.) evoked by each stimulus paradigm normalized to [DA]_o_ evoked by a single orange Lstim in DLS (**d**, *n* = 5 in *N* = 3 animals) and NAcc (**f**, *n* = 5 in *N* = 4 animals). *P = 0.0183, ****P < 0.0001, two-sided one-sample *t*-test versus single orange Lstim before adding DHβE. Horizontal dashed lines indicate peak [DA]_o_ evoked by a single stimulus of same color data as a reference. Source data

**Extended Data Fig. 2:**
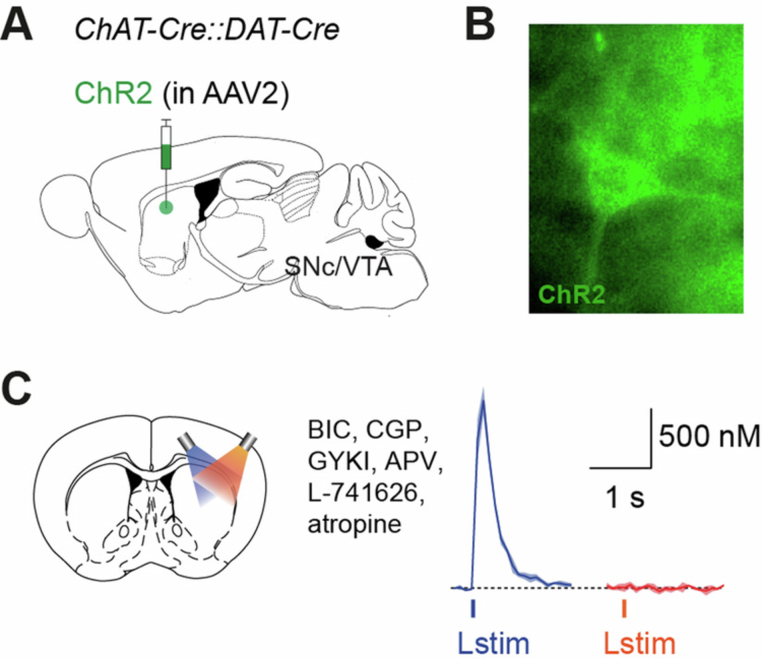
Orange LED protocol does not trigger detectable DA release. **a**, Schematic of virus injection. **b**, A Chl expresses ChR2 (scale bar, 20 µm). **c**, *Left*, Schematic of stimulating configuration. *Right*, mean transients from representative experiments of [DA]_o_ ( ± SEM) evoked by a single pulse of blue Lstim (*blue line*), or orange Lstim (orange *line*). Blue light activated ChR2-eYFP-expressing ChIs to drive DA release. Orange light did not drive observable DA release via Chls. Antagonists present for GABA_A_ (bicuculline, BIC), GABA_B_ (CGP 55845), AMPA (GYKI 5246), NMDA (D-APV), D_2_ (L-741,626) and mACh (atropine) receptors. Source data

To demonstrate further that stimulation of ChIs depresses subsequent DA_DA_ release (and to further avoid confounding effects on presynaptic short-term plasticity of DA or ACh release probabilities resulting from summation of stimuli), we used a dual optogenetic approach in double-transgenic ChAT-Cre:DAT-Cre mice to tailor stimulation to ChIs versus DA axons, in DLS and NAcc. ChR2 packaged in AAV2 (for anterograde axonal transport)^14^ was injected to the striatum for preferential expression in ChIs and to minimize expression in DA axons (Fig. 2a,b). Chrimson (packaged in AAV5) was injected in substantia nigra pars compacta (SNc) or ventral tegmental area (VTA) for expression in DA neurons and axons (Fig. 2a,b). In the striatum, we used a minimal intensity of blue light (480 nm laser, 2 ms) to stimulate ChIs preferentially without driving detectable [DA]_o_, and a threshold level orange light (585 ± 22 nm light-emitting diode (LED), 2 ms) to preferentially stimulate DA axons and drive detectable [DA]_o_ (DA_DA_) (Fig. 2c,e and Extended Data Fig. 2), in the presence of a cocktail of antagonists for GABA, AMPA, NMDA, D_2_ and mACh receptors. After minimal blue light stimulation of ChIs, [DA]_o_ evoked by subsequent orange light stimulation of DA axons 25 ms afterwards was significantly lower compared with [DA]_o_ evoked by an orange light pulse alone, in both DLS (Fig. 2c,d) and NAcc (Fig. 2e,f). Furthermore, this attenuation of light-evoked [DA]_o_ by prior ChI stimulation was prevented by antagonizing nAChRs with DHβE (Fig. 2c–f). Therefore, activation of nAChRs at a level below the threshold for driving DA release can profoundly depress subsequent DA_DA_ through a mechanism unrelated to changes to availability of DA vesicles.

**Fig. 3:**
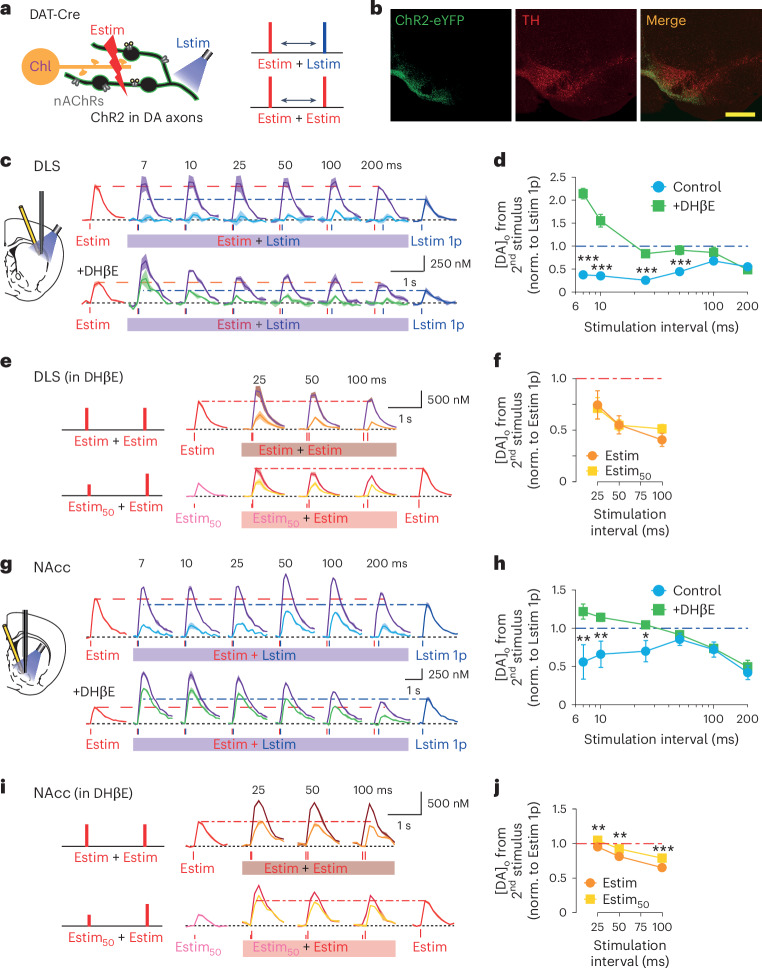
Activation of ChIs depresses subsequent DA release evoked by optogenetic stimulation. **a**, Schematic of stimulation configuration for **c**–**j**. Local electrical stimulation (Estim) in striatal slices and blue light stimulation (Lstim) of ChR2-eYFP-expressing DA axons. **b**, ChR2-eYFP expression in midbrain DA neurons co-immunoreactive for DAT in DAT-Cre mice, after example VTA injection. Scale bar, 400 µm. **c**,**g**, Mean transients from representative experiments of [DA]_o_ (± s.e.m.) evoked by a single pulse of Estim (red lines) or Lstim (blue lines), or paired Estim plus Lstim pulses (purple) at ISI of 7–200 ms in DLS (**c**) and NAcc (**g**) when nAChRs can be active (no DHβE) (top) or when nAChRs are antagonized (DHβE present) (bottom) in DAT-Cre mice. The light-blue trace shows the [DA]_o_ attributable to the paired Lstim after subtraction of [DA]_o_ due to Estim without DHβE. The green trace shows the [DA]_o_ attributable to the paired Lstim in the presence of DHβE. **d**,**h**, The mean peak [DA]_o_ (± s.e.m.) evoked by the paired Lstim normalized to [DA]_o_ evoked by a single Lstim, versus ISI in DLS (**d**, *N* = 5 animals) and NAcc (**h**, *N* = 5 animals). *P < 0.05, **P < 0.01, ***P < 0.001, two-way ANOVA with Fisher’s LSD test post hoc. **e**,**i**, Mean transients from representative experiments of [DA]_o_ (± s.e.m.) evoked by a single full-strength Estim (red), paired Estims (brown) or a single low-intensity Estim (Estim_50_, pink) with paired full-strength Estim (dark red) at ISIs of 25–100 ms in DLS (**e**) and NAcc (**i**) in wild-type animals. The orange and yellow traces show the [DA]_o_ attributable to the paired Estims after subtraction of [DA]_o_ due to Estim 1p. **f**,**j**, The mean peak [DA]_o_ (± s.e.m.) evoked by the paired stimulations versus ISI in DLS (**f**, *N* = 5 animals) and NAcc (**j**, *N* = 5 animals). DHβE was present in **e**, **f**, **i** and **j**. **P < 0.01, ***P < 0.001, two-way ANOVA with Fisher’s LSD test post hoc. **c**,**e**,**g**,**i**, Horizontal dashed lines indicate peak [DA]_o_ evoked by a single stimulus of same color data as a reference. Source data

We then used an alternative stimulation paradigm to characterize the time course of ChI-dependent depression of subsequent DA_DA_ release. We combined an initial electrical stimulus, which drives DA_DA_ + DA_ChI_, with a subsequent targeted optogenetic stimulation of DA_DA_, by activating DA axons expressing ChR2 with blue light in ex vivo striatal slices from DAT-Cre mice (Fig. 3a,b) in DLS (Fig. 3c,d) and NAcc (Fig. 3g,h) and examined the difference in depression seen when β2-nAChRs were available versus antagonized (DHβE, 1 µM). [DA]_o_ evoked by the subsequent light pulse was depressed compared with [DA]_o_ evoked by a single light pulse alone and for interstimulus intervals up to ~100 ms (DLS) or ~50 ms (NAcc). This depression was relieved when nAChRs were antagonized. The relief from depression was not a consequence of less DA depletion arising from the lower levels of DA release evoked by initial electrical stimulation: in wild-type mice, we halved the level of initial DA release to a level similar to that seen for a single pulse (1p) in the presence of DHβE, by lowering electrical stimulation intensity (Estim_50_) but did not find a corresponding increase in the [DA]_o_ evoked by a subsequent full intensity electrical stimulation, which evoked the same [DA]_o_ as after a full-strength stimulus in DLS (Fig. 3e,f), and only slightly greater [DA]_o_ in NAcc (Fig. 3i,j). Rather, the activation of nAChRs by ChIs depresses DA release during subsequent activation of DA axons through a mechanism independent of prior ACh or DA release that is more pronounced and longer lasting in DLS than NAcc, for durations that are notably similar to the duration of ChI pauses.

### Activation of nAChRs limits subsequent increase in axonal Ca^2^ and membrane depolarization

**Fig. 4:**
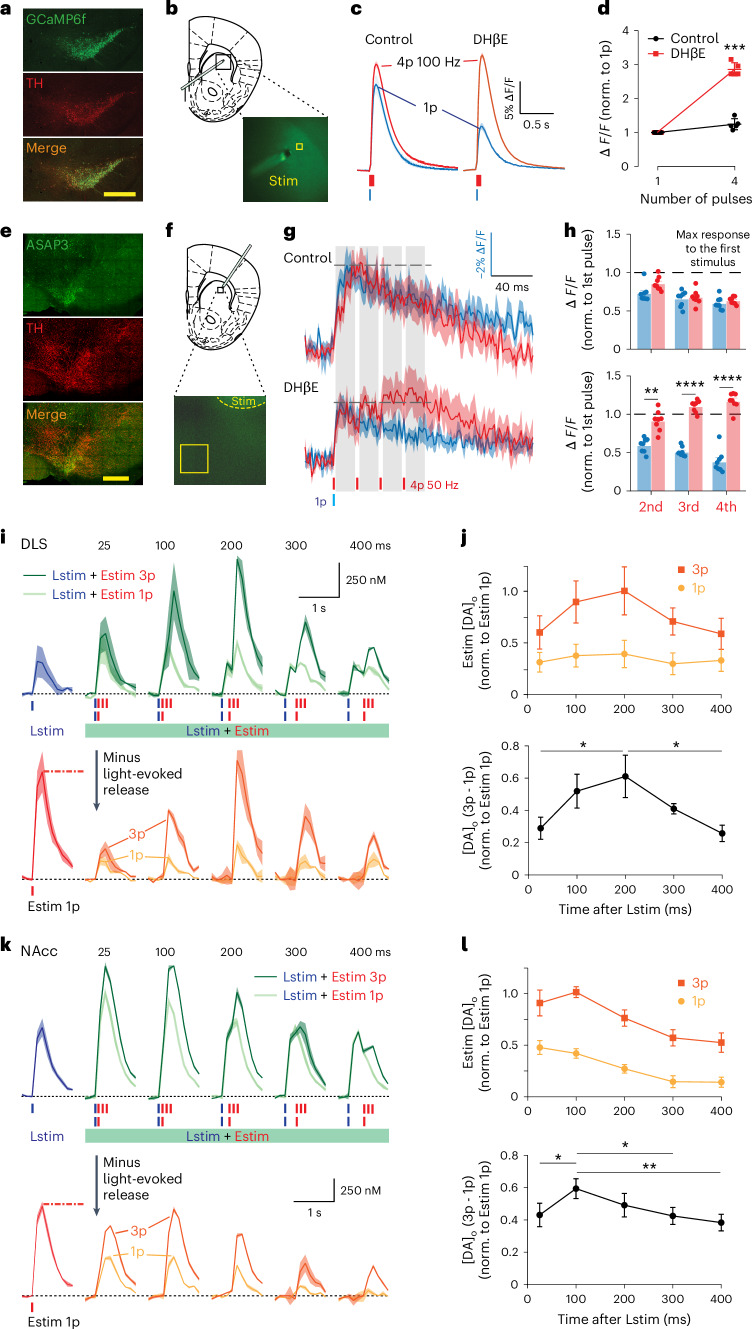
Activation of ChIs and nAChRs attenuates repetitive axonal depolarization and calcium summation in DA axons, and at longer intervals limits nAChR control of DA release. **a**, Images of VTA and SNc from DAT-Cre:Ai95D mice showing GCaMP6f-eGFP expression (green) in TH‐immunopositive neurons (red) (scale bar, 400 µm). **b**, An illustration of stimulation configurations in live tissue. **c**, Examples of Ca^2+^‐imaging responses (changes to GCaMP6f fluorescence, Δ*F*/*F*) (mean ± s.e.m. from duplicates) in a DA axon population imaged in DLS in response to single pulses or trains of four electrical pulses at 100 Hz in control conditions (left) and in the presence of DHβE (1 μM) (right). **d**, Mean peak values (± s.e.m.) for GCaMP6f Δ*F*/*F* versus pulse numbers. The data are normalized to value for one pulse (*N* = 5 animals). ***P = 0.0004 two-sided paired *t*-test [DA]_o_ from four pulses of stimulation. **e**, Images of VTA and SNc from DAT-Cre mice with ASAP3 expression (green) and TH-positive neurons (red) (scale bar, 400 µm). **f**, An illustration of stimulation configuration in live tissue. **g**, Averaged transients of voltage sensor (changes to ASAP3 fluorescence, −Δ*F*/*F*, mean ± s.e.m.) in striatal DA axons to single (blue) or four electrical pulses at 50 Hz (red) before (top) and after (bottom) antagonizing nAChRs with DHβE. The scale bar vertical axis applies to 1p data only. For the four-pulse data, the maximum value generated after the first pulse of stimulation and occurring before the second pulse is scaled to match the peak value seen after a 1p stimulation (*n* = 8 recordings in *N* = 4 animals). **h**, The peak value (mean ± s.e.m.) observed in the averaged ASAP3 responses after successive pulses in the train (gray in **g**) before and after DHβE, normalized to the maximum value seen after the first pulse (*n* = 8 recordings in *N* = 4 animals). **P = 0.0055, ****P < 0.001, one-way ANOVA with the Geisser–Greenhouse correction and Šidák’s multiple-comparisons tests. **i**,**k**, Mean transients from representative experiments of [DA]_o_ (± s.e.m.) evoked by a single pulse of Lstim (blue) or Estim (red lines), or Lstim paired with either one (1p) or three (3p) Estim pulses (green) at ISIs of 25–400 ms spanning activation, desensitization and resensitization in DLS (**i**) and NAcc (**k**) in the striatum of ChAT-Cre:Ai32 mice. The yellow and orange traces show the [DA]_o_ attributable to the paired stimuli after subtraction of Lstim. **j**,**l**, Top: mean peak [DA]_o_ (± s.e.m.) evoked by paired Estims for 1p (yellow) and 3p (orange) normalized to [DA]_o_ evoked by a single Estim, versus ISI, for DLS (**j**, *N* = 5 animals) and NAcc (**l**, *N* = 5 animals). Bottom: differences between [DA]_o_ evoked by 3p and 1p Estims. *P < 0.05, **P < 0.01, repeated-measures one-way ANOVA with two-sided Tukey test post hoc. Source data

DA release is strongly governed by axonal activation mechanisms upstream of Ca^2+^ entry, as well as those governing Ca^2+^ entry, intracellular buffering and signaling^13,15–17^. To understand the depression of DA release that follows initial activation of striatal nAChRs, we first tested whether nAChR activation also limited subsequent axonal Ca^2+^ influx at successive stimuli. In brain slices of DAT-Cre:Ai95D mice (Fig. 4a,b), we imaged Ca^2+^ reporter GCaMP6f in DA axons in DLS as previously^13^, after single versus four-pulse electrical stimuli (100 Hz, or 143 Hz corresponding to 7 ms IPI), with and without nAChR antagonism. DA release evoked by this protocol is very sensitive to nAChR activation^2,18^: the ratio of [DA]_o_ evoked by a 100 Hz pulse train versus single pulses is only a little over 1 when nAChRs are activated but is markedly increased when nAChRs are antagonized. In parallel, the ratio of axonal GCaMP6f fluorescence for a train compared with a single pulse was only slightly greater than 1 when nAChRs were active (Fig. 4c,d and Extended Data Fig. 4) but markedly greater than 1 when we antagonized β2-nAChRs (DHβE, 1 µM) (Fig. 4c,d and Extended Data Fig. 4), indicating that nAChR activation limits the summation of axonal [Ca^2+^]_i_ during subsequent stimuli. Absolute levels were not compared before and after DHβE because of the propensity for signal decay/bleaching over time and the nonlinear relationships between [Ca^2+^]_o_ and neurotransmitter release^19^.

**Extended Data Fig. 3:**
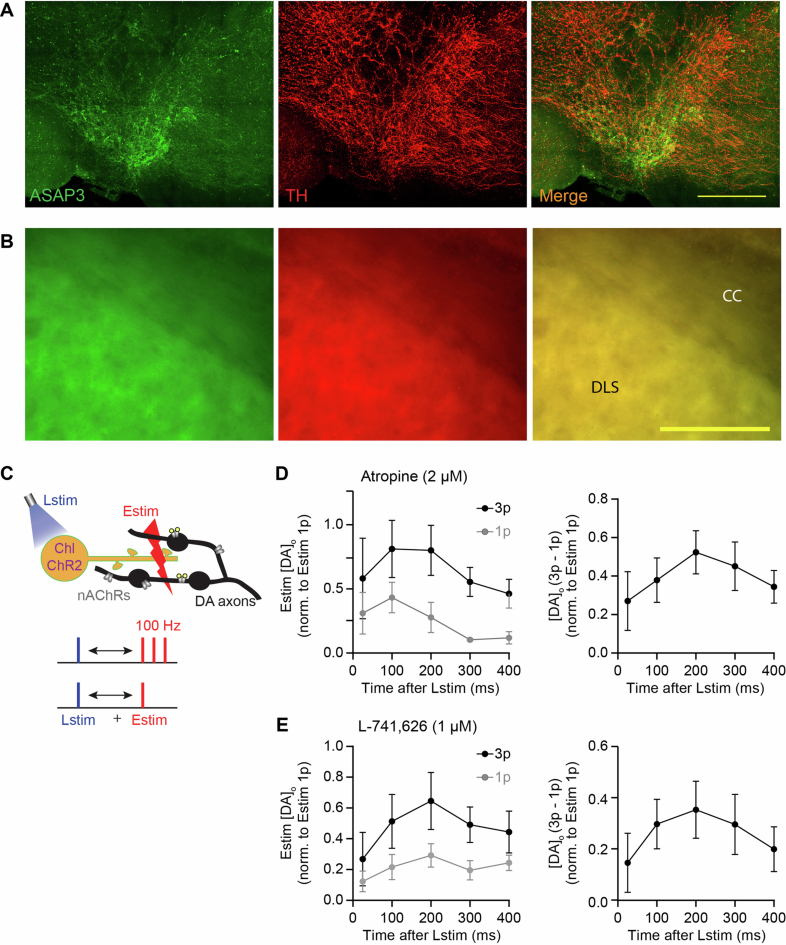
Expression of ASAP3 in DA neurons, and dynamic changes to release of DA after light activation of ChIs (100-400 ms interval) persist in DLS in the presence of antagonists for either mAChRs or D_2_ receptors. **a**, Expression of ASAP3-GFP in DA neurons after viral injection within midbrain, co-labelled for TH-immunoreactivity (scale bar, 400 µm). **b**, The co-expression of ASAP3 and TH in the DLS. CC: corpus callosum (scale bar, 400 µm). **c**, Schematic of stimulation configuration used in D and E. Blue light stimulation (*Lstim*) of ChR2-eYFP-expressing ChIs, subsequent local electrical stimulation (*Estim*) in striatal slices from ChAT-Cre:Ai32 mice. **d, e**, *Left*, mean peak [DA]_o_ ( ± SEM) evoked in DLS after a Lstim by a subsequent Estim of either 1p (*grey*) or 3p 100 Hz (*black*) at a range of ISI, normalised to [DA]_o_ evoked by a single Estim, after antagonising either muscarinic receptors with atropine (2 µM) (D, n = 5 recordings, N = 3 animals) or D_2_ receptors with L-741,626 (1 µM) (E, n = 5 recordings, N = 4 animals). *Right*, differences between [DA]_o_ evoked by 3p and 1p Estims. Significant effect of IPI seen in bell-shaped curves for both, P < 0.001, One-way ANOVA. Source data

**Extended Data Fig. 4:**
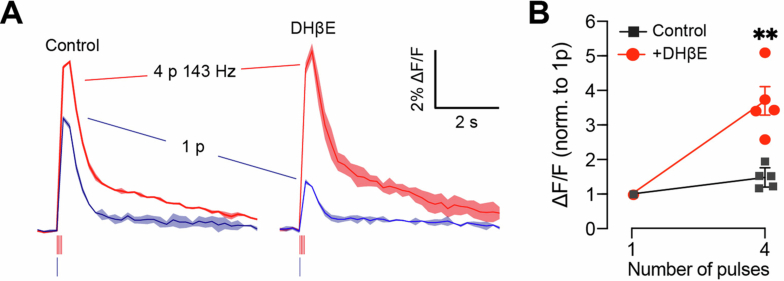
nAChRs limit summation of intracellular calcium in DA axons during a train of electrical stimulation ex vivo. **a**, Examples of GCaMP6f fluorescence **signals**, ΔF/F (mean ± SEM from duplicates) in a DA axon population imaged in **DLS** ***ex vivo*** in response to single or trains of 4 electrical pulses (4p) at 143 Hz (7 ms inter-stimulation interval) in control conditions (left) and in the presence of DHβE (1 μM) (right). **b**, Mean peak values (± SEM) for GCaMP6f ΔF/F vs. pulse numbers. Data are normalised to value for 1 pulse (n = 5 recordings, N = 2 animals). **P = 0.0025 two-sided paired t-test [DA]_o_ from 4 pulses of stimulation. Source data

To test whether nAChRs directly limit axonal depolarization during successive stimulation of DA axons, we imaged the ASAP3 voltage sensor^20^ expressed in DA axons in DLS in brain slices after single versus four-pulse electrical stimuli (50 Hz), with and without nAChR antagonism (Fig. 4e,f and Extended Data Fig. 3). In control conditions in the absence of a nAChR antagonist, a subsequent depolarization event could be detected in DA axons in response to a pulse train that closely matched the depolarization after a single pulse (Fig. 4g,d), whereas when nAChRs were antagonized, the depolarization response detected during a train of pulses could be differentiated from that seen for a single pulse, by successive depolarization events (Fig. 4g,h). These results indicate that the activation of nAChRs initially depolarizes DA axons, in agreement with other reports^21^, but in consequence, for a time window of ~50–100 ms this leads to an extended refractory-like period, when subsequent depolarization and Ca^2+^ entry are limited, with further DA release prevented.

### nAChRs activated by initial excitation are off during ChI rebound

In an in vivo multiphasic response, ChIs show burst–pause activity followed by a rebound increase in activity some ~100–300 ms after initial excitation^22–24^. At these intervals, the ChI-dependent depression of DA release from the initial excitation will have dissipated (Fig. 1). However, for the ensuing ~1 s, it has been shown that further DA release is resistant to targeted optogenetic stimulation of ChIs but not DA axons^4,11^. This selective refractoriness of DA release to activation of ChIs but not DA axons suggests that nAChRs cannot be activated by ACh at the time of rebound ChI activity. One potential explanation could be the desensitization of nAChRs that follows their activation^25–27^ and has been suspected to play a role in DA release dynamics during stimulus trains^28^. We tested whether the DA release dynamics at the time points of ChI rebound activity are consistent with the lack of nAChR activation for example by nAChR desensitization, by exploiting the observations that whereas the activation of nAChRs limits DA release at subsequent pulses within 100 ms (as in Fig. 1), the desensitization or inactivation of nAChRs allows subsequent release during a high-frequency train and increases the ratio of [DA]_o_ evoked by trains versus single pulses^2^. In striatal slices from ChAT-Cre:Ai32 mice, we light-activated ChIs, then 25–400 ms later applied either single or triplets of electrical pulses (100 Hz) to explore how the difference in [DA]_o_ evoked by triplet versus single pulses varied with time (Fig. 4i,k and Extended Data Fig. 3c). The difference in [DA]_o_ evoked by triplet versus single pulses varied with interval, peaking at ~200 ms in DLS and ~100 ms in NAcc, and decaying by 400 ms (Fig. 4i–l). Neither muscarinic nor D_2_-receptor activation was responsible for these dynamics (Extended Data Fig. 3d,e). These dynamics probably reflect the initial inability and then renewed ability to activate nAChRs, which could reflect the time course of nAChR desensitization and desensitization. They indicate that nAChRs will not be in an activatable state at ChI rebound activity ~100–300 ms after initial excitation and will not then be strongly limiting or driving DA release at this interval. The regional differences in time courses parallels differences in α4β2-nAChR stoichiometries^29,30^ that might contribute to different desensitization time courses.

### nAChR antagonism in vivo promotes DA release, DA axon activity and induces conditioned place-preference

**Fig. 5:**
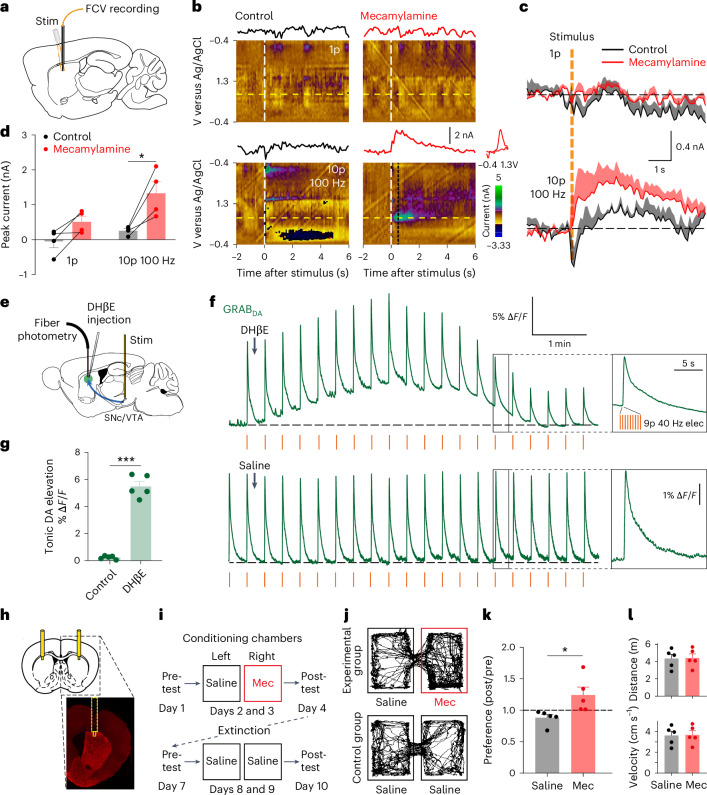
nAChR antagonism in DLS in vivo promotes DA release and conditioned place preference. **a**, Schematic of configuration of FCV recording and stimulation electrodes in DLS. **b**, Color plots and representative line plots of oxidation current (from dashed yellow row at approximately +0.7 V) of voltammetric DA current versus time and a corresponding DA cyclic voltammogram (from time point of dotted black line) before and after mec (i.p.). **c**, DA signals (mean ± s.e.m.) evoked by one pulse or ten pulses (10p) at 100 Hz before (black) and after (red) mec (2 mg kg^−1^ i.p.). **d**, Mean peak DA currents (± s.e.m.). **P = 0.0340 for two-sided Šidák’s post-hoc test with multiple comparisons adjustment (*N* = 4 animals). **e**, The schematic of configuration of optic-fiber photometry recording in DLS and stimulation in SNc. **f**, Example traces of fiber photometry recording of GRAB_DA2m_ signal with local injection of DHβE (0.07 µg in 200 nl saline) or saline (200 nl) showing an elevation in baseline levels of non-evoked [DA]_o_ after DHβE only. Intermittent electrical stimulation (nine pulses (9p) at 40 Hz elec, red lines) of SNc was applied at 0.05 Hz as a reference. **g**, The mean maximal change in baseline tonic DA (± s.e.m.) after local injection of DHβE or saline control. ***P = 0.0001, two-sided paired *t*-test (*n* = 5 from *N* = 3 animals). **h**, Schematic illustration of a bilateral cannula system for local infusion to dorsal striatum, and an example hemislice with DAPI staining. **i**, Conditioning paradigm. Mice were conditioned for four times over 2 days (20 min per session) after local infusion of mec (10 µg per side, right chamber, red) or saline (0.5 µl over 1 min, left chamber, black), whereas the control groups received saline for both chambers. **j**, Representative tracking traces from the postconditioning day. **k**, Preference for the right chamber after conditioning (± s.e.m.). *P = 0.0451, two-sided paired *t*-test, *N* = 5 animals in each test. **l**, Total travel distances and velocity of movements (± s.e.m.) in open-field test (*N* = 5 animals). Source data

In vivo, ChIs fire tonically at 3–10 Hz, which leads to an ongoing background level of nAChR activation and desensitization that governs DA. Desensitization of nAChRs might continuously limit how nAChRs suppress DA output. We tested in vivo whether nAChRs are able to depress DA release or are desensitized. In wild-type urethane-anesthetized mice, we found that [DA]_o_ evoked in DLS by local electrical stimulation with brief pulse trains and detected using FCV was increased after systemic injection of nAChR antagonist mecamylamine (mec, Fig. 5a–d), indicating that nAChR activation is limiting DA release in vivo. Similarly, previous work in NAcc has shown that systemic nAChR antagonist increases DA release in response to reward, in freely moving rats^31^.

**Extended Data Fig. 5:**
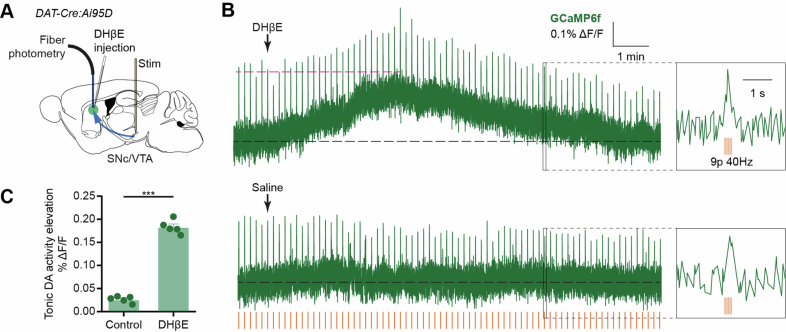
Local antagonism of striatal nAChRs enhances axonal calcium in DA axons *in vivo.* **a**, Schematic of configuration of fibre photometry recording in DLS and stimulation in SNc. **b**, Example traces of fibre photometry recording of GCaMP6f signal with local injection of DHβE (0.07 µg in 200 nl saline) or saline (200 nl). Electrical stimulation (9 pulses at 40 Hz, red lines) was applied at 0.1 Hz in SNc to gain a reference amplitude. Insets, show expanded example signals. **c**, Mean maximal increase in baseline non-evoked GCaMP6f signal in DA axons (‘tonic DA activity’) ( ± SEM) after local injection of DHβE or saline. ***P < 0.0001 two-sided paired t-test (n = 5 from N = 3 animals). Source data

Furthermore, we explored the impact of local antagonism of β2-nAChRs in DLS on levels of DA release detected in vivo using genetically enocded fluorescent DA sensor GRAB_DA2m_ (G-protein-receptor-based sensor DA_2m_) (ref. ^32^) in wild-type mice, and separately, on DA axon activity reported by GCaMP6f in DAT-Cre:Ai95 mice, using optic-fiber photometry in urethane-anesthetized mice. Signals typically revealed a bleaching over time that led to rundown of evoked signal amplitudes (midbrain stimulation 9 pulses, 40 Hz, 10 s intervals), and therefore, evoked levels were not quantified but served instead as a reference for changes to nonevoked signals. Given that ChIs and DA neurons are spontaneously active in vivo (tonic 3–10 Hz activity plus phasic burst events) without external stimulation, we postulated that ChIs might be inhibiting DA release levels under nonevoked conditions. Indeed, local striatal injection of DHβE (0.07 µg in 200 nl) significantly increased the amplitude of nonevoked levels of fluorescence of GRAB_DA2m_ (Fig. 5e–g) and axonal GCaMP6f (Extended Data Fig. 5) in absolute terms and compared with saline controls (despite the underlying signal bleaching over time) by amplitudes comparable to signals evoked by midbrain stimulation. Therefore, in vivo, ChIs acting at nAChRs are continuously operating a suppression of DA axon activity and DA release during endogenous activity in ChIs and DA neurons.

We then tested in freely moving mice whether nAChRs in dorsal striatum could modify reward-related learning as might be predicted for modulation of DA function^33^. After 2 days of daily conditioning, mice developed a conditioned place preference for the chamber conditioned with intrastriatal diffusion in DLS of nAChR antagonist mecamylamine but not saline controls (Fig. 5h–l). Our findings are consistent with in vivo studies in adjacent NAc showing that nAChR antagonists increase reward-evoked DA levels and promote reward-related learning^31,34^.

### Tonic and multiphasic activity in ChIs depresses DA release in a computational model

**Fig. 6:**
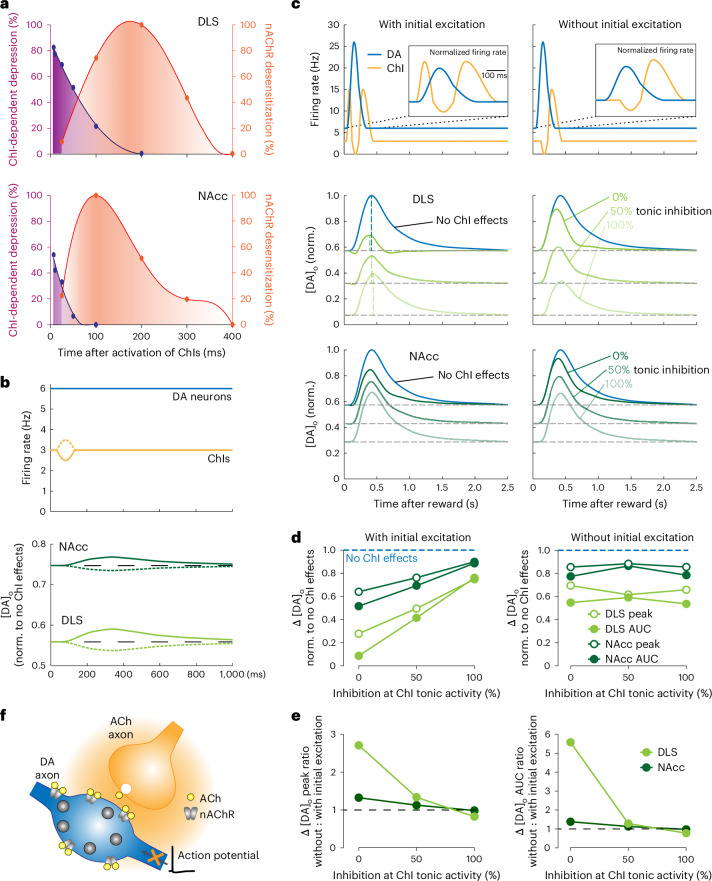
ChI-dependent attenuation of striatal DA release dominates in a computational model. **a**, Values used in the model for the strength of ChI-dependent depression of DA release (purple) and the normalized level of apparent nAChR desensitization (orange) versus time after ChI activation in DLS (top) and NAcc (bottom). **b**, When the firing rate of DA neurons (blue) is constant, a brief decrease (solid) or increase (dotted) of ChI activity (yellow) can respectively increase or decrease [DA]_o_ in DLS (light green) and NAcc (dark green). **c**, Top row: multiphasic ChI responses (yellow) with (left) or without (right) initial excitation phases, plus DA neuron burst activity (blue) from ref. ^23^ were inputted to predict striatal DA release (bottom rows) in DLS and NAcc when the background tonic level of ChI-dependent depression of DA release was set to 0% (green), 50% (lighter green) and 100% (lightest green). Insets: The firing rates of DA and ChI neurons normalized to their own tonic firing frequencies. **d**, Summary of peak [DA]_o_ (open circle) and area under [DA]_o_ curve (circle) in DLS (light green) and NAcc (dark green) with (left) and without (right) initial excitation in ChI multiphasic activity, when ChI-dependent attenuation was set to 0%, 50% and 100% at tonic activity of ChIs. **e**, The ratio of peak [DA]_o_ (left) and area under [DA]_o_ curve (right) release when ChIs without and with initial excitation. **f**, Schematic showing that activation of ChIs and β2*-nAChRs on DA axons limits action potential propagation. AUC, area under the curve.

**Extended Data Fig. 6:**
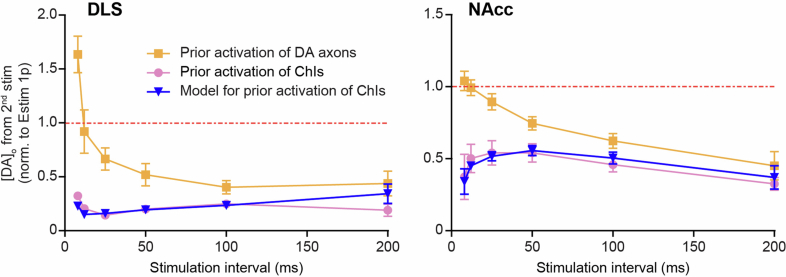
The computational model successfully predicts empirical *ex vivo* data. The computational model built from empirical data **from** Fig. 3 D,H predicted the impact of prior activation of ChIs on subsequent DA release ( ± SEM) by an electrical stimulation as in Fig. 1d, j. Because the electrical stimulation (the second stimulation in Fig. 1d, h) evoked DA_DA_ and DA_ChI_ rather than only the DA_DA_ evoked by light stimulation (the second stimulation in Fig. 3d, h), the predicted data (*blue*) was scaled as a whole curve to match the overall mean of the experimental data (*pink*) in DLS (*left*) and NAcc (*right*) to overcome the unknown ChI-induced inhibition of DA_ChI_ in this experimental paradigm (N = 5 animals). Source data

We developed a computational model to predict how nAChRs impact on [DA]_o_ in vivo in DLS and NAcc during the dynamic tonic and multiphasic activity in ChIs that is coincident with phasic activity in DA neurons (Fig. 6a). The model incorporates the timings of the dynamic suppression of DA release by ChIs (from Fig. 3), and the decay (desensitization) and recovery of nAChR control of DA release (from Fig. 4), as well as the extracellular kinetics of DA signals (detected after electrical stimulation when nAChRs are off; Fig. 1). We validated that the model could simulate ex vivo observations of Fig. 1d,j (Extended Data Fig. 6).

**Extended Data Fig. 7:**
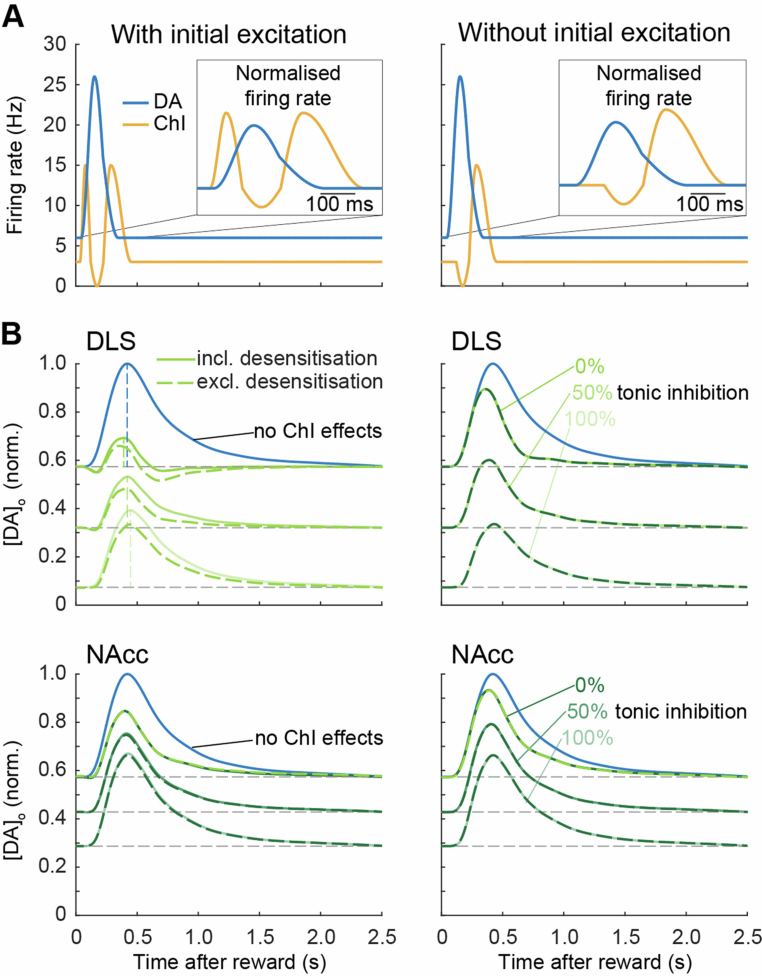
‘Desensitization’ response of nAChRs plays a limited role in modifying phasic DA release in a computational model during multiphasic ChI activity. **a**, Coincident phasic DA neuron firing (*blue*) and multiphasic ChI activity (*yellow*) with (*left*) or without (*right*) initial excitation in ChIs. Inset, firing rates normalized to baseline rates. **b**, Phasic DA release was modified by ChI activity (compared to no ChI effects, *blue*) both with (*solid green line*) and without (*dashed green line*) a ‘desensitization’ component in the model (obtained from Fig. 4j,l) for DLS (*upper*) and NAcc (*lower*). Background levels of tonic suppression of DA output by ChIs included in the model were 0, 50 and 100%. Inclusion of a dynamic desensitization-like component in the model impacted on phasic DA release only when there was initial ChI excitation (*left)*, and only in DLS, acting to slightly increase phasic DA release by preventing nAChRs from inhibiting DA release during a rebound activity phase in ChIs. The effect is prominent in DLS (*upper*) than in NAcc (*lower*), consistent with the stronger ChI-induced inhibition of DA release in DLS than NAcc. As expected, excluding a dynamic desensitization-like component from the model did not affect DA release when the initial excitation was absent because there was no initial increase in nAChR activation. Source data

We first explored how changes to ChI tonic firing rate might modulate tonic levels of [DA]_o_ in the absence of underlying changes in DA neuron firing rate. We set the background level of ChI-induced suppression of DA release to an arbitrary value of 50% of the maximum observed in each region, and normalized [DA]_o_ to the level seen without ChI effects. The model illustrates how transient (~100 ms) multiphasic changes in ChI activity generate opposite changes in [DA]_o_ (Fig. 6b). This illustration helps to explain the observations in vivo that [DA]_o_ can be modified without an underlying change to DA neuron firing rate^6^. We then tested how multiphasic ChI activity modifies [DA]_o_ during concurrent burst activity in DA neurons^23^, both with and without the initial excitation that occurs in half of ChIs^22^ (Fig. 6c). We incorporated a range of background levels of tonic ChI-induced depression of DA release (0%, 50% and 100%) before multiphasic activity, which correspondingly reduced the baseline level of DA release (Fig. 6c). In response to DA neuron burst activity, DA release was lowered by concurrent multiphasic activity in ChIs, and to a greater extent (1) when multiphasic activity included initial ChI excitation, (2) when background level of ChI-induced depression of DA release was at a lower initial percent strength against which the effect of multiphasic activity was offset, and (3) in DLS than in NAc (Fig. 6c–e). The strongest predicted reduction in burst DA release arose particularly from the initial excitation in ChIs on a minimal prior background of ChI activity. In the absence of initial excitation, the strongest reduction in DA release resulted from the depression of late DA release by ChI rebound activity. A nAChR desensitization-like component played only a minor role in these outcomes, and in DLS more than NAc, and only when initial excitation was present (Extended Data Fig. 7). Overall, the model suggests that multiphasic activity in ChIs attenuates DA release by phasic activity in DA neurons, particularly in DLS and particularly after initial excitation in ChIs.

**Extended Data Fig. 8:**
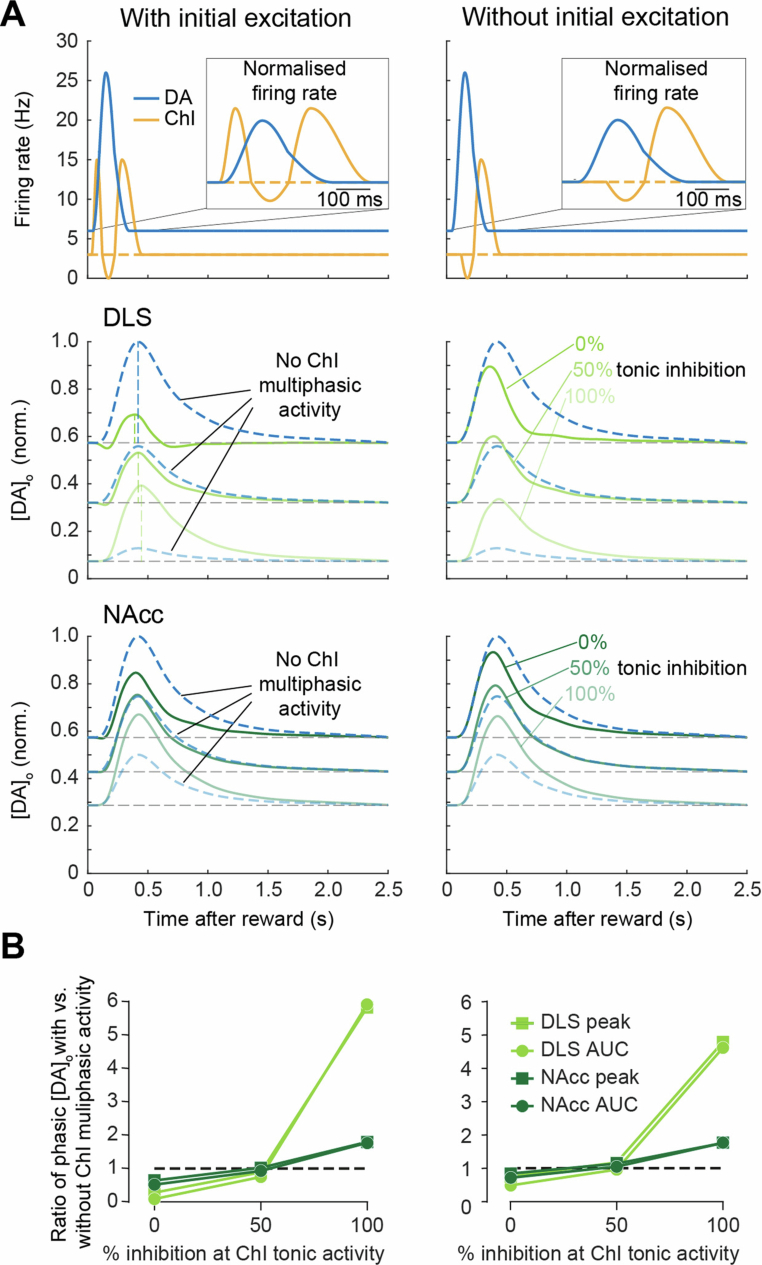
The tonic level of ChI-induced depression of DA release modifies the relative impact of ChI multiphasic activity on phasic DA release in a computational model. **a**, *Upper*, Model for coincident phasic DA neuron firing (*blue*) and multiphasic ChI activity (*yellow*) with (*left*) or without (*right*) initial excitation in ChIs. *Lower panels*, comparison of phasic DA signals at three different background levels of tonic suppression of DA output (0, 50, 100%) before (*dashed blues*) versus after (*solid greens*) the development of multiphasic activity. At low-intermediate levels of tonic ChI-induced suppression of DA release ( ≤ 50%), multiphasic ChI activity reduced phasic DA release, whereas at very high levels of tonic ChI-induced suppression of DA release (50–100%), ChI multiphasic activity enhanced phasic DA release. **b**, Summary of impact of multiphasic ChI activity on phasic DA release on different background levels of tonic suppression of DA release by tonic ChI activity. Change to phasic DA signal amplitude and area under curve (AUC) with (*left*) or without (*right*) a phase of initial ChI excitation. Source data

The model also indicated that tonic ChI activity in the absence of multiphasic ChI activity, reflective of scenarios before learning^22,35^, reduces levels of DA release during tonic and burst activity in DA neurons (Extended Data Fig. 8a, blue lines). Incorporation of multiphasic ChI activity on a low-intermediate background level of suppression of DA release by ChIs (<50%) further reduced phasic DA release. Only at extremely high background levels of suppression (50–100%) did ChI multiphasic activity actually enhance phasic DA release (Extended Data Fig. 8a,b), owing to relief of ChI-mediated depression of DA release enabled by a ChI pause. Therefore, the level of phasic striatal DA release is a dynamic function of both tonic and multiphasic activity in ChIs.

## Discussion

Cholinergic interneurons have become a major focus of interest owing to their potential to regulate DA output. Here, we reveal that the activation of ChIs and nAChRs and the consequent depolarization of the DA axon, at levels below (or above) those required to drive DA release, is followed by a strong refractory-like suppression of the subsequent excitability of DA axons, preventing further depolarization by subsequent arriving activity, and limiting axonal Ca^2+^ summation and DA release. Our combined ex vivo, in vivo and in silico approaches together show that, during physiological activity in vivo, ChIs then operate a strongly limiting effect that inversely scales DA release during activity in DA neurons. These findings support a major role for axonal integration as a mechanism to prevent neurotransmitter output and also necessitate revision of a range of current prevailing views about how ACh governs DA output, in the following ways.

### Dynamic refractoriness of DA axons versus instantaneous depolarization of DA axons

The activation of ChIs can rapidly depolarize DA axons via activation of nAChRs and ectopic action potential generation^7,36^ to drive instantaneous DA release^4,5^. However, recent studies have not found evidence that ChIs drive or change the timing of DA release in vivo^8–10^. Our data reveal that there are other powerful outcomes of nAChR activation. We show that DA release is not the most feasible outcome of activation of ChIs/nAChRs in vivo, as it sits within an opposing context of ongoing ACh–DA integration. We show that activity in ChIs, after initial activation of nAChRs and axonal depolarization, leads to an extended refractoriness of DA axons and to subsequent activation by stimuli arriving up to ~100 ms later. This could be mediated through either depolarization-dependent ion channel inactivation or a form of shunting inhibition during or resulting from nAChR channel conductances. This striking refractoriness to further depolarization provides an ongoing interruption of the relationship between spikes occurring in DA neurons and DA release to diminish the amplitude of DA output according to recent ChI activity.

We show that ChIs can more easily limit than activate DA release: ChIs can profoundly depress DA release after levels of ChI activation that are less than those required to drive detectable instantaneous DA release. Therefore, in vivo, when ChI activity is ongoing but also less synchronized than after artificial stimulations^4,5^, ChIs and ACh release are more likely to reach the lower levels that are sufficient to inhibit DA release than the higher levels required to trigger it. Indeed, in vivo we confirmed that the net outcome of activating nAChRs is to depress, and not drive, DA release. These interpretations do not exclude the possibility that activation of ChIs might also trigger DA release in certain scenarios in vivo. For example, if sufficient interval has lapsed since nAChR activation so that the subsequent suppression of DA axon activity is minimized, nAChRs are recovered from their activation–desensitization cycle and a population of ACh boutons or ChIs then fire in synchrony, a large instantaneous increase in ACh release and nAChR activation might result and be sufficient to trigger DA release in some microdomain. This might be more likely in NAc where the depression of DA release is weaker than DLS. Correspondingly, the relationships between concurrent ACh and DA signaling might show localized variation that can also vary over time.

Nonetheless, our data in vivo from detection of DA using GRAB_DA2m_ sensor and axonal activity (using calcium imaging) show that activity in ChIs in vivo predominantly depresses DA release. These data, in conjunction with our modeling of DA release under different levels of activity in ChIs and DA neurons, show that the impact of ChI activity on DA release varies dynamically with tonic and multiphasic activity in ChIs and provides a continuously dynamic and inverse scaling factor on the amplitude of DA signals.

### Depression due to refractoriness versus depletion of vesicle pool

We identified that the limitation on subsequent DA release after nAChR activation was not due to a potential depletion of the DA vesicle pool that might follow an initial release event^11,12^. We saw that a low-level stimulation of ChIs that did not result in detectable DA release and could not have depleted the DA vesicle pool nonetheless led to a profound depression of subsequent DA release, whether the subsequent stimulus was applied to ChIs or DA axons (Figs. 1–3). Therefore, the refractoriness on DA release after ChI activation does not occur via depletion of the DA vesicle pool.

### Refractoriness of subsequent DA release versus firing frequency-dependent filtering

The finding that nAChR activation limits subsequent DA axon depolarization is not equivalent to the previous theory that ChIs provide a frequency filter on DA release in which the modulation of DA release by nAChRs varies with DA neuron firing frequency^2,37^. The interpretation of a filtering effect was based on the observation that, during concurrent activation of DA axons and ChIs, nAChRs appeared to support DA release driven by trains of pulses at low but not high frequencies, akin to a form of low-frequency-pass filter. Our new observations now reveal that this apparent frequency filtering is not in fact intrinsically related to the processing of frequency of DA neuron activity by DA axons but rather will be an outcome of the refractoriness of DA axons to any stimulus arriving within ~100 ms of nAChR activation. With concurrent local stimulation of DA axons and ChIs, DA release is restricted to the initial summed DA_DA_ + DA_ChI_, with little ensuing release for any stimuli arriving up to ~100 ms later, leading to minimal apparent sensitivity of DA release to subsequent DA neuron activity arriving in this brief time window, that is, for short trains at frequencies ≥10 Hz. The apparent low-frequency pass filtering effect in those experiments was an inadvertent outcome of the synchronized activation of DA axons and ChIs and the dynamic timecourse of suppression of DA release after nAChR activation. Here, we reveal that this outcome of ChIs on DA release does not necessarily or intrinsically vary with the frequency of DA neuron firing per se, contrary to previous hypotheses. Rather, the impact of ChIs on preventing DA release depends on the interval between activity in ChIs and any subsequent activity in DA neurons, whether low or high frequency. Because, in vivo, ChIs typically fire tonically at 3–10 Hz plus intermittent bursts, and each DA release site is likely to be under the control of a network of ChIs, ChIs probably provide a continuous (if dynamic) inhibition of subsequent DA release that scales more with the intensity and time since recent activity in the ChI network rather than DA firing frequency.

The underlying frequency dependence of DA release will continue to be sculpted by other intrinsic mechanisms that regulate short-term facilitation or depression of DA release probability, with short-term depression mechanisms that operate independently of nAChR action persisting over much longer timescales (several seconds)^13^ than the refractoriness of release operated by nAChRs (~100 ms).

### Attenuation versus enhancement of phasic DA release

Synchronized activation of a small population of ChIs by optogenetic or electrical activation or stimulation of their cortical or thalamic inputs has previously been shown to directly drive DA release^4,5,11,12,38^, leading to speculations that the initial excitation or rebound activity in multiphasic ChI activity acquired in vivo in response to rewards or a reward-related cues during reinforcement learning drives DA release. Our combined observations ex vivo and in vivo suggest that, by contrast, ChIs operate a predominantly limiting inverse scaling effect on DA release.

In addition, the pause in a ChI multiphasic response has previously been speculated to promote coincident burst-evoked DA, because high-frequency stimuli can induce more DA release when nAChRs are turned off^2,39^. However, while a reduction in ChI activity from tonic levels or after an initial phase of excitation in a multiphasic burst may relieve the inhibition on DA release, its attenuation for up to 100 ms will persist into a short pause phase and continue to place some limitations on DA release. This effect will decay during the pause and scale with prior ChI activity and will be more profound with greater initial excitation. Our model also shows that, although ChIs inversely regulate the amplitude or scale of DA signals, they do not modify their kinetics.

### Regional heterogeneity of ChI-induced depression of DA release

ChIs prevent DA axonal depolarization more strongly and for longer durations in DLS than in NAcc, which leads to critical distinctions in function. DA neurons encode reward predictions and their errors through phasic firing frequency but despite largely similar events in SNc^1^ and VTA^40^ during learning, there are larger-amplitude DA release events detected in ventral (from VTA) than dorsal (from SNc) striatum in vivo^41,42^. This discrepancy does not occur ex vivo, where local striatal stimulations with single-pulse stimuli typically evoke lower [DA]_o_ in NAc than DLS^18,43^, indicating that a dynamic circuit(s) found in vivo is more permissive for phasic DA release in NAc than DLS. The lesser refractoriness of DA release after ChI/nAChR activity in NAc than in DLS is a candidate explanation (Fig. 6b,c) and, in turn, suggests that ChI-induced depression of DA release may play a critical role in regulating how reward prediction errors translate differently to DA release in different striatal regions^42,44,45^ and, thus, contribute to striatal learning^46^.

In summary, activation of striatal β2-nAChRs and nAChR-mediated depolarization of DA axons for endogenous levels of activity predominantly leads to an apparent refractoriness of DA axons, which transiently depresses action-potential-mediated depolarization and DA release. This mechanism operates dynamically on DA axons according to ChI activity to dominate as a gain mechanism that inversely scales DA output according to the recent history of ChI activity, continuously and dynamically.

## Methods

Details of key lab materials used and generated in this study are listed in a Key Resources Table in ref. ^47^. Protocols associated with this work can be found at ref. ^48^.

### Animals

Mice used in ex vivo experiments and in vivo DA recordings were male and female adult wild-type C57BL/6J mice (Charles River) (21–40 days), heterozygous ChAT-Cre:Ai32 (6–16 weeks), heterozygous DAT-Cre:Ai95D (4–7 weeks), heterozygous DAT-Cre:ChAT-Cre mice (8–12 weeks) or heterozygous DAT-IRES-Cre (B6.SJL-Slc6^a3tm1.1(cre)Bkmn^/J, JAX stock number 006660) (8–16 weeks) injected with viral vectors. Heterozygote ChAT-Cre:Ai32 mice were generated from ChAT-Cre^+/+^ mice (B6;129S6-Chat^tm2(cre)Lowl^/J, JAX stock number 006410) crossed with Ai32^+/+^ mice (B6;129S-Gt(ROSA) 26Sor^tm32(CAG-COP4*H134R/EYFP)Hze/^J, JAX stock number 012569). Heterozygote DAT-Cre:Ai95D mice were generated from DAT-IRES-Cre^+/−^ mice crossed with Ai95D^+/+^ mice (B6:129S-Gt(ROSA)26Sor^tm95.1(CAG-GCaMP6f)Hze^/J). Male-only C57BL/6N mice (Charles River) (42–50 days) were used for behavioral experiments.

Animals were group-housed and maintained at 20–24 °C and 45–65% humidity on a 12-h light/dark cycle with ad libitum access to food and water. The procedures for ex vivo recordings and anesthetized in vivo DA recordings were performed in accordance with the Animals in Scientific Procedures Act (UK) 1986 (amended 2012) with ethical approval from an Animal Welfare and Ethical Review Body at the University of Oxford, and under authority of project licence P9371BF54 granted by the UK Home Office. Behavioral experiments were performed using protocols approved by the Animal Care and Use Committees at the Chinese Institute for Brain Research (CIBR-IACUC-007) and were performed in accordance with the guidelines established by the US National Institutes of Health.

### Virus injection

Mice were placed in a stereotaxic frame under isoflurane anesthesia, and a craniotomy was made above the injection site. Injections of 1 μl virus were given unilaterally or bilaterally in VTA (coordinates from bregma in mm: anterior-posterior (AP) −3.1, medial-lateral (ML) ±0.5, dorsal-ventral (DV) −4.4), SNc (AP −3.5, ML ±1.2, DV −4.0), DLS (AP +1.0, ML ±2.0, DV −1.8) or NAcc (AP +1.0, ML ±1.1, DV −3.8) using a 2.5 μl 33-gauge Hamilton syringe at 0.2 µl min^−1^ with a microinjector. The syringe was left in place for 5 min after each injection, then retracted slowly. Animals were maintained for at least 3 weeks after surgery to allow virus expression in the striatum.

For expression of ChR2 or ASAP3 in DA axons, heterozygote DAT-IRES-Cre mice were intracranially injected in SNc/VTA with a Cre-inducible recombinant AAV serotype 5 vector containing an inverted gene for either channelrhodopsin-2 fused in-frame with a gene encoding enhanced yellow fluorescent protein (pAAV5-hEF1α-DIO-hChR2(H134R)-EYFP-WPRE-pA) (titer 1 × 10^12^ vg ml^−1^, University of North Carolina Vector Core) or fluorescent voltage sensor ASAP3 without the Kv soma-targeting signal (AAV5-EF1α-DIO-ASAP3-WPRE) (titer 2.4 × 10^12^ vg ml^−1^, Stanford Gene Vector and Virus Core).

For dual optogenetic experiments, heterozygote DAT-Cre:ChAT-Cre mice were intracranially injected in midbrain with a Cre-inducible recombinant AAV serotype 5 vector containing an inverted gene for Chrimson (ssAAV-5/2-hEF1α/hTLV1-dlox-ChrimsonR-tdTomato(rev)-dlox-WPRE-bGHp(A), v288-5 ETH Zurich) and in the striatum with a Cre-inducible recombinant AAV serotype 2 vector containing an inverted gene for channelrhodopsin-2 fused in-frame with a gene encoding enhanced yellow fluorescent protein (pAAV-double floxed-hChR2(H134R)-EYFP-WPRE-pA) (titer 1 × 10^12^ vg ml^−1^, University of North Carolina Vector Core).

For expression of GRAB_DA2m_, wild-type C57BL/6J mice were intracranially injected in dorsal striatum with AAV serotype 5 vector containing GRAB_DA2m_ (AAV5-hSyn-GRABDA2m (titer 1 × 10^13^ vg ml^−1^, BrainVTA).

### Ex vivo slice voltammetry and stimulation

For FCV in acute coronal slices, animals were anesthetized with isoflurane. Brains were quickly removed into ice-cold, high-Mg^2+^ cutting solution containing in mM: 85 NaCl, 25 NaHCO_3_, 2.5 KCl, 1.25 NaH_2_PO_4_, 0.5 CaCl_2_, 7 MgCl_2_, 10 glucose, 65 sucrose. Brains were then blocked, and 300 µm coronal slices were cut on a vibratome (Leica VT1200S) between +1.5 and +0.5 mm from bregma containing caudate putamen and nucleus accumbens. Slices recovered at 32 °C for 30–40 min after dissection and were subsequently kept at room temperature. Slices were maintained and recorded in artificial cerebrospinal fluid (aCSF) containing in mM: 130 NaCl, 25 NaHCO_3_, 2.5 KCl, 1.25 NaH_2_PO_4_, 2.5 CaCl_2_, 2 MgCl_2_, 10 glucose. The aCSF was saturated with 95% O_2_/5% CO_2_; recordings were made at 32–33 °C. Extracellular DA concentration ([DA]_o_) was measured using FCV with 7-µm-diameter carbon fiber microelectrodes (tip length 50–100 µm) and a Millar voltammeter (Julian Millar, Barts and the London School of Medicine and Dentistry) as previously. The voltage was applied as a triangular waveform (−0.7 to +1.3 V range versus Ag/AgCl) at a scan rate of 800 V s^−1^, and data were sampled at 8 Hz.

For optogenetic stimulations, ChR2-expressing ChIs or DA axons were activated using a 473-nm diode laser (DL-473, Rapp Optoelectronic) coupled to the microscope with a fiber optic cable (200 µm multimode, numerical aperture (NA) 0.22). Spot illumination had a 30 µm diameter under a 40× immersion objective. Laser pulses were 2 ms duration, 5–23 mW mm^−2^ at specimen. Chrimson-expressing DA axons were activated using a LED with a 585 ± 22 nm filter. LED pulses were 2 ms duration, 2.5–3.4 mW mm^−2^. The Lstim_0_ was achieved by lowering the laser intensity to the point at which there was no detectable evoked FCV signal above noise, in on-line or off-line analyses (Extended Data Fig. 1).

For electrical stimulations, 0.65 mA current (200 µs width) was delivered through a surface bipolar concentric Pt/Ir electrode (125 µm outer diameter, 25 µm inner diameter) (FHC) placed ~100 µm from the recording electrode. The Estim_50_ was the stimulation current at which evoked [DA]_o_ was ~50% of that seen with normal stimulation (0.65 mA) during on-line analysis. Stimulations were timed to avoid FCV scans.

### Calcium imaging ex vivo

As in our previous study^13^, an Olympus BX51Wl microscope equipped with a OptoLED Lite system (CAIRN Research), Prime Scientific CMOS (sCMOS) Camera (Teledyne Photometrics) and a 40×/0.8 NA water objective (Olympus UK) was used for wide-field fluorescence imaging of GCaMP6f in dopaminergic axons in DLS in ex vivo slices in response to electrical stimulus pulses given singly and or in trains of four pulses at 100 Hz. Images were acquired at 16.6 Hz frame rate every 2.5 min using Micro-Manager 1.4, with stimulation and recording synchronized using custom-written procedures in Igor Pro 6 (WaveMetrics) and an ITC-18 A/D board (Instrutech). Image files were analyzed with MATLAB R2019b and Fiji 1.5. We extracted fluorescence intensity from the region of interest of 25 µm × 25 µm, which was 50 µm away from the electrical stimulating electrode tip. Ca^2+^ transients were bleach-corrected by fitting an exponential curve function through both the baseline (2 s before stimulation) and the last 1 s in a 7.2 s recording window. The order of single and train stimulations was alternated and equally distributed, and data were collected in duplicate before and after a change in extracellular experimental condition. Data are expressed as Δ*F*/*F*, where *F* is the fitted curve.

### Voltage sensor imaging ex vivo

An Olympus BX51Wl microscope equipped with a OptoLED Lite system (CAIRN Research), an iXon EMCCD Camera (ANDOR) and a x0×/0.8 NA water objective (Olympus) was used for wide-field imaging of ex vivo slices in response to electrical stimulus pulses, given singly or in four-pulse train (50 Hz). Images were acquired at 660 Hz frame rate every 2.5 min using Micro-Manager 1.4, with stimulation and recording triggered using PClamp. Image files were analyzed with MATLAB R2019b and Fiji 1.5. We extracted fluorescence intensity from the region of interest (~5 µm × 5 µm). ASAP3 transients were bleach-corrected by fitting an exponential curve function. The order of single and train stimulations was alternated and equally distributed, and data were collected in duplicate before and after a change in extracellular experimental condition. Observations were time-locked to the deflection. Data are expressed as Δ*F*/*F*, where *F* is the fitted curve.

### In vivo recordings under anesthesia

Wild-type and transgenic mice were anesthetized with urethane (1.4–1.9 g kg^−1^, intraperitoneal (i.p.); Biolab), supplemented with additional urethane (0.2 g kg^−1^) every 1–2 h as required. All wounds and pressure points were infiltrated with bupivacaine (0.5%). Upon reaching surgical anesthesia, the head was fixed in a stereotaxic frame (Kopf). Core temperature was maintained at 35–36 °C using a homeothermic blanket and monitored via a rectal probe (TR-100, Fine Science Tools). Mecamylamine (2 mg kg^−1^) was injected intraperitoneally to block nAChRs in the striatum.

### In vivo voltammetry

A round piece of skull overlying the left hemisphere was removed to target the DLS (from bregma: AP +1.0 mm, ML ±1.6 mm, DV −2.2 mm). A stimulating and recording array consisting of a carbon-fiber microelectrode and a bipolar stimulating electrode (MS303/3-A/SPC, P1 Technology) was positioned in the DLS. The Ag/AgCl reference electrode was implanted in another part of the forebrain. [DA]_o_ was measured using FCV with 7-µm-diameter carbon fiber microelectrodes (tip length 50–100 µm) and a Tarheel system (University of Washington). The voltage was applied as a triangular waveform (−0.4 to +1.3 V range versus Ag/AgCl) at a scan rate of 400 V s^−1^, and data were sampled at 10 Hz. The location of the tip of FCV electrode was confirmed histologically. For striatal electrical stimulation, 0.65 mA current (200 µs) was delivered through a bipolar stimulating electrode (0.005 inch, MS303/3-A/SPC, P1 Technologies). The stimulating electrode tips were separated by ~500 µm and were glued to the FCV recording electrode to fix the tip of the FCV electrode between the two stimulating poles.

### In vivo fiber photometry

Round pieces of skull overlying the left hemisphere were removed to allow access to the DLS (from bregma: AP +1.0 mm, ML ±1.6 mm, DV −2.2 mm) and SNc (AP −3.1 mm, ML ±0.8 mm, DV −4.3 mm). The injection and recording array, consisting of a glass pipette and a 200-µm-diameter fiber, was positioned in the DLS. GCaMP6f expressed in DA axons or GRAB_DA2m_ was activated with 480 nm light (76 µW), and the intensity of GCaMP6f or GRAB_DA2m_ emission was sampled at 40 Hz with Neurophotometrics (FP3001). For midbrain electrical stimulation, 0.5 mA current (500 µs) was delivered through a bipolar stimulating electrode (0.005 inch, tip separation ~500 µm, MS303/3-A/SPC, P1 Technologies) at 0.1 Hz (GCaMP experiments) or 0.05 Hz (GRAB_DA_ experiments).

### Behavioral recordings

#### Cannula placements

Male C57BL/6N mice (42–50 days old) were anesthetized with isoflurane (5% induction, 1.5–2% maintenance) and placed on a stereotaxic frame for surgery. Bilateral injection needles (outer diameter 0.21 mm, inner diameter 0.11 mm) (RWD Life Science) with the guide cannula (outer diameter 0.41 mm, inner diameter 0.25 mm) (RWD Life Science) were implanted to the dorsal striatum either vertically or at a small angle from the vertical, with the tip of each cannula aimed at the following coordinates: AP +1.0 mm; ML ±1.6 mm to bregma; DV −2.4 mm (from dura). Mice recovered for 3 days after surgery.

#### Conditioned place preference testing

In CPP experiments, mice were placed in a 40 cm × 40 cm transparent plexiglass arena that was divided into two equal chambers separated by a doorway. The chambers were decorated with either horizontal or vertical stripes. The movement of animals was recorded and analyzed with Smart V3.0 tracking software (Panlab). On day 1, mice were allowed to freely shuttle between two chambers to assess place preference at baseline, expressed as percentage of time spent in the right chamber. The mice were conditioned on days 2 and 3, when animals received alternating bilateral striatal injection with either mecamylamine (10 µg per side) or saline vehicle (0.9%) in a volume of 0.5 µl over 1 min in AM and PM. Animals were then constrained respectively in the right or left chamber for 20 min. The treatments were counterbalanced for time of day. On day 4, the postconditioning chamber preference was calculated as the percentage of time spent in the right mecamylamine-associated chamber compared with on preconditioning day 1. For the next two days (days 5 and 6), animals received bilateral saline injection and explored both chambers for 20 min after which place preference was extinguished. The conditioning procedure was then repeated but for bilateral saline for both chambers, with a preconditioning test on day 7, 2 days of conditioning on days 8 and 9 and a postconditioning test on day 10. To minimize place preference bias at baseline, the five animals in each test showing the least place preference on the preconditioning day (mecamylamine 42–58%, control 45–55%) were selected for subsequent conditioning. For open-field experiments, mice received bilateral striatal injection of either saline vehicle or mecamylamine (10 µg per side), and were placed into the open field chamber to assess total running distance and average velocity within 20 min.

### Immunocytochemistry

After voltammetry recordings in acute slices, slices were fixed in 4% paraformaldehyde (PFA) dissolved in PBS containing 0.2% picric acid. Slices were fixed overnight at 4 °C and then stored in PBS. Free-floating sections were then washed five times in PBS for 5 min and incubated in 0.5% Triton X-100 and 10% normal donkey serum.

ChIs expressing ChR2-eYFP were identified as ChAT-immunoreactive as previously^24^. Fixed and rinsed slices were incubated overnight with 1:100 goat anti-ChAT antibody (AB144P, Sigma Aldrich) or for 5 days with 1:200 goat anti-ChAT antibody (SAB2500233, Sigma Aldrich) dissolved in PBS containing 0.5% Triton X-100 and 3% normal donkey serum. Sections were then washed five times with PBS for 5 min and incubated for 2 h at room temperature with 1:1000 Alexa Fluor 568 donkey anti-goat (A-11057, Thermo Fisher Scientific) or AMCA-conjugated donkey anti-goat secondary antibody (705-155-147, Jackson Immuno Research Labs) dissolved in PBS containing 0.5% Triton X-100 and 3% normal donkey serum.

DA neurons coexpressing ChR2-eYFP, GCaMP6f-eYFP, ASAP3 or Chrimson and striatal DA axons were identified by immunoreactivity to tyrosine hydroxylase (TH) as previously^13^. Fixed and rinsed slices were incubated overnight with 1:2,000 rabbit anti-TH antibody (Sigma Aldrich) dissolved in PBS containing 0.5% Triton X-100, 1% normal goat serum and 1% fetal bovine serum. Sections were then washed five times with PBS for 5 min and incubated for 2 h at room temperature with 1:1,000 DyLight 594 goat anti-rabbit (Abcam) or CoraLite488 goat anti-rabbit secondary antibody (Proteintech) dissolved in PBS containing 0.5% Triton X-100, 1% normal goat serum and 1% fetal bovine serum.

Sections processed as above were then washed with PBS and mounted on gelled slides with Vectashield mounting medium (Vector Labs) and imaged at 20×/0.8 NA, using a Zeiss LSM880 confocal microscope system running ZEN black version 2.3 (Zeiss), or on a confocal microscope system (FV1000 IX81, Olympus) using a 20×/0.75 NA objective and Fluoview software (Olympus). Maximum intensity projection from a *z*-stack of height 30 µm was captured individually and the stack of the pictures were compressed. Red fluorescence (TH and ChAT) was captured at 638–759 nm with 633 nm excitation. Green fluorescence (GCaMP, ChR2 and ASAP3) was captured at 493–630 nm with 488 nm excitation.

To verify carbon-fiber locations in dorsal striatum for in vivo FCV recordings, anesthetized mice were euthanized and brains were quickly removed and fixed in 4% PFA overnight. The fixed brains were then sectioned into 50-μm slices using a vibratome (Leica). Slices were rinsed with PBS three times, mounted on glass slides and then imaged under microscope to identify the location of recording sites.

To verify placements of intrastriatal injection cannulae in behavioral experiments, mice were anesthetized by i.p. injection of sterile Avertin (250 mg kg^−1^ body weight) and transcardially perfused with saline and 4% PFA. Brains were dissected and postfixed overnight in 4% PFA then dehydrated by 30% sucrose for 24 h. The fixed brains were then frozen-sectioned into 50-μm slices using a vibratome (Leica). To verify the placement of cannulae, slices were stained with immunoreactivity to TH as slices from ex vivo experiments and mounted on glass slides. The slices were imaged under inverted confocal microscope (Zeiss) with a 405-nm laser for excitation.

### Drugs

DHβE and mecamylamine hydrochIoride for ex vivo and anesthetized in vivo experiments were purchased from Tocris Bioscience. Mecamylamine hydrochIoride for behavioral experiments were purchased from Sigma-Aldrich. All other chemicals were purchased from Sigma-Aldrich. Pharmacological drugs for ex vivo experiments were prepared in distilled deionized water as stock aliquots at 1,000× final concentrations and stored at −20 °C. Drug stocks were then diluted to the final concentration in carbogenated aCSF immediately before use and were bath-applied. Drugs for in vivo experiments were dissolved in sterilized saline to the final concentration.

### Computational model

The computational model was written in MATLAB and is available via GitHub at https://github.com/craggASAP/Axonal_Brake.git. The model included the following: (1) the dynamic strength of ChI-dependent depression, determined from the ratio of [DA]_o_ evoked at a second stimulus before and after antagonizing β2*-nAChRs with DHβE (from Fig. 3d,h; DAT-Cre, light stimulus) fitted to a polynomial curve (DLS: *y* = 1.97 × 10^5^*x*^2^ − 0.00833*x* + 0.872, *R*^2^ = 0.99; NAcc: *y* = 8.71 × 10^5^*x*^2^ − 0.0149*x* + 0.611, *R*^2^ = 0.96); (2) the profile of apparent nAChR desensitization (Fig. 4j,l) estimated from the change in the difference between [DA]_o_ evoked by three pulses and one pulse at a second stimulus (from Fig. 4i,k), normalized to a maximum and fitted with polynomial curves. DLS: *y* = 2 × 10^−5^*x*^2^ − 0.0087*x* + 0.88, NAcc: *y* = −9 × 10^−10^*x*^4^ + 9 × 10^−7^*x*^3^ − 0.0003*x*^2^ + 0.039*x* − 0.5654; and (3) the dynamic release and uptake profile of [DA]_o_ seen after a single electrical stimulation ex vivo to model [DA]_o_ in vivo as a scalar product with DA neuron activity. We also included in the model a range of potential levels of background ChI-dependent suppression of DA release (0%, 50% and 100%) arising from tonic activity in ChIs.

We excluded a potential component of DA release that can be driven by synchronized activation of ChIs in some experimental scenarios (Fig. 1)^4,5^, because we found that the threshold for nAChR-mediated suppression of DA release is lower than that required to drive release ex vivo (Fig. 1) and was met in vivo after discrete striatal stimulation (Fig. 4). We also excluded short-term depression arising from nAChR-independent mechanisms, as this covaries only minimally on the short and rapid timescales relevant to the timescale of multiphasic activities^13^.

### Statistics and reproducibility

Statistical analysis was performed using GraphPad Prism v7. Sample sizes were chosen on the basis of our previous work with similar techniques. No data were excluded from the analyses. The investigators were not blinded to allocation during experiments and outcome assessment. Experimental sequences were randomized where possible. Data are expressed as mean ± s.e.m. Images are representative images from at least three independent replications. The *N* value is the number of animals, and the *n* value is the number of individual recordings. A range of tests were used as specified.

### Reporting summary

Further information on research design is available in the Nature Portfolio Reporting Summary linked to this article.

## Online content

Any methods, additional references, Nature Portfolio reporting summaries, source data, extended data, supplementary information, acknowledgements, peer review information; details of author contributions and competing interests; and statements of data and code availability are available at 10.1038/s41593-025-01906-5.

## Supplementary information

Reporting Summary

## Source data

Source Data Figs. 1–5 Numerical data points used for plots and statistical analyses.

Source Data Extended Data Figs. 1–8 Numerical data points used for plots and statistical analyses.

## Data Availability

Data used in this study are available via Zenodo at 10.5281/zenodo.13898624 (ref. ^47^). Source data are provided with this paper.

## Code availability

The code used study is available via GitHub at https://github.com/craggASAP/Axonal_Brake.git and via Zenodo at 10.5281/zenodo.14622173 (ref. ^49^).

## References

[1] Schultz, W., Dayan, P. & Montague, P. R. A neural substrate of prediction and reward. *Science* **275**, 1593–1599 (1997). 9054347 10.1126/science.275.5306.1593

[2] Rice, M. E. & Cragg, S. J. Nicotine amplifies reward-related dopamine signals in striatum. *Nat. Neurosci.* **7**, 583–584 (2004). 15146188 10.1038/nn1244

[3] Sulzer, D., Cragg, S. J. & Rice, M. E. Striatal dopamine neurotransmission: regulation of release and uptake. *Basal Ganglia* **6**, 123–148 (2016). 27141430 10.1016/j.baga.2016.02.001 PMC4850498

[4] Threlfell, S. et al. Striatal dopamine release is triggered by synchronized activity in cholinergic interneurons. *Neuron* **75**, 58–64 (2012). 22794260 10.1016/j.neuron.2012.04.038

[5] Cachope, R. et al. Selective activation of cholinergic interneurons enhances accumbal phasic dopamine release: setting the tone for reward processing. *Cell Rep.* **2**, 33–41 (2012). 22840394 10.1016/j.celrep.2012.05.011 PMC3408582

[6] Mohebi, A. et al. Dissociable dopamine dynamics for learning and motivation. *Nature* **570**, 65–70 (2019). 31118513 10.1038/s41586-019-1235-y PMC6555489

[7] Liu, C. et al. An action potential initiation mechanism in distal axons for the control of dopamine release. *Science* **375**, 1378–1385 (2022). 35324301 10.1126/science.abn0532 PMC9081985

[8] Krok, A. C. et al. Intrinsic dopamine and acetylcholine dynamics in the striatum of mice. *Nature* **621**, 543–549 (2023). 37558873 10.1038/s41586-023-05995-9 PMC11577287

[9] Chantranupong, L. et al. Dopamine and glutamate regulate striatal acetylcholine in decision-making. *Nature* **621**, 577–585 (2023). 37557915 10.1038/s41586-023-06492-9 PMC10511323

[10] Azcorra, M. et al. Unique functional responses differentially map onto genetic subtypes of dopamine neurons. *Nat. Neurosci.* **26**, 1762–1774 (2023). 37537242 10.1038/s41593-023-01401-9 PMC10545540

[11] Wang, L. et al. Modulation of dopamine release in the striatum by physiologically relevant levels of nicotine. *Nat. Commun.* **5**, 3925 (2014). 24968237 10.1038/ncomms4925

[12] Wang, L. et al. Temporal components of cholinergic terminal to dopaminergic terminal transmission in dorsal striatum slices of mice. *J. Physiol.* **592**, 3559–3576 (2014). 24973407 10.1113/jphysiol.2014.271825 PMC4229348

[13] Condon, M. D. et al. Plasticity in striatal dopamine release is governed by release-independent depression and the dopamine transporter. *Nat. Commun.* **10**, 4263 (2019). 31537790 10.1038/s41467-019-12264-9 PMC6753151

[14] Ciesielska, A. et al. Anterograde axonal transport of AAV2-GDNF in rat basal ganglia. *Mol. Ther.* **19**, 922–927 (2011). 21102559 10.1038/mt.2010.248 PMC3098627

[15] Brimblecombe, K. R. et al. Calbindin-D28K limits dopamine release in ventral but not dorsal striatum by regulating Ca^2+^ availability and dopamine transporter function. *ACS Chem. Neurosci.* **10**, 3419–3426 (2019). 31361457 10.1021/acschemneuro.9b00325 PMC6706870

[16] Brimblecombe, K. R., Gracie, C. J., Platt, N. P. & Cragg, S. J. Gating of dopamine transmission by calcium and axonal N-, Q-, T- and L-type voltage-gated calcium channels differs between striatal domains. *J. Physiol.* **593**, 929–946 (2015). 25533038 10.1113/jphysiol.2014.285890 PMC4398530

[17] Kramer, P. F., Twedell, E. L., Shin, J. H., Zhang, R. & Khaliq, Z. M. Axonal mechanisms mediating gamma-aminobutyric acid receptor type A (GABA-A) inhibition of striatal dopamine release. *eLife* **9**, e55729 (2020). 32870779 10.7554/eLife.55729 PMC7462615

[18] Threlfell, S. et al. Striatal muscarinic receptors promote activity dependence of dopamine transmission via distinct receptor subtypes on cholinergic interneurons in ventral versus dorsal striatum. *J. Neurosci.* **30**, 3398–3408 (2010). 20203199 10.1523/JNEUROSCI.5620-09.2010 PMC2866006

[19] Johnson, S. L., Marcotti, W. & Kros, C. J. Increase in efficiency and reduction in Ca^2+^ dependence of exocytosis during development of mouse inner hair cells. *J. Physiol.* **563**, 177–191 (2005). 15613377 10.1113/jphysiol.2004.074740 PMC1665557

[20] Villette, V. et al. Ultrafast two-photon imaging of a high-gain voltage indicator in awake behaving mice. *Cell* **179**, 1590–1608.e23 (2019). 31835034 10.1016/j.cell.2019.11.004 PMC6941988

[21] Kramer, P. F. et al. Synaptic-like axo-axonal transmission from striatal cholinergic interneurons onto dopaminergic fibers. *Neuron* **110**, 2949–2960.e44 (2022). 35931070 10.1016/j.neuron.2022.07.011 PMC9509469

[22] Aosaki, T. et al. Responses of tonically active neurons in the primate’s striatum undergo systematic changes during behavioral sensorimotor conditioning. *J. Neurosci.* **14**, 3969–3984 (1994). 8207500 10.1523/JNEUROSCI.14-06-03969.1994 PMC6576948

[23] Morris, G., Arkadir, D., Nevet, A., Vaadia, E. & Bergman, H. Coincident but distinct messages of midbrain dopamine and striatal tonically active neurons. *Neuron* **43**, 133–143 (2004). 15233923 10.1016/j.neuron.2004.06.012

[24] Zhang, Y. F., Reynolds, J. N. J. & Cragg, S. J. Pauses in cholinergic interneuron activity are driven by excitatory input and delayed rectification, with dopamine modulation. *Neuron* **98**, 918–925 (2018). 29754751 10.1016/j.neuron.2018.04.027 PMC5993868

[25] Dani, J. A. & Bertrand, D. Nicotinic acetylcholine receptors and nicotinic cholinergic mechanisms of the central nervous system. *Annu. Rev. Pharmacol. Toxicol.* **47**, 699–729 (2007). 17009926 10.1146/annurev.pharmtox.47.120505.105214

[26] Changeux, J. P., Devillers-Thiery, A. & Chemouilli, P. Acetylcholine receptor: an allosteric protein. *Science* **225**, 1335–1345 (1984). 6382611 10.1126/science.6382611

[27] Giniatullin, R., Nistri, A. & Yakel, J. L. Desensitization of nicotinic ACh receptors: shaping cholinergic signaling. *Trends Neurosci.* **28**, 371–378 (2005). 15979501 10.1016/j.tins.2005.04.009

[28] Shin, J. H., Adrover, M. F. & Alvarez, V. A. Distinctive modulation of dopamine release in the nucleus accumbens shell mediated by dopamine and acetylcholine receptors. *J. Neurosci.* **37**, 11166–11180 (2017). 29030431 10.1523/JNEUROSCI.0596-17.2017 PMC5688525

[29] Exley, R. et al. Distinct contributions of nicotinic acetylcholine receptor subunit α4 and subunit α6 to the reinforcing effects of nicotine. *Proc. Natl Acad. Sci. USA* **108**, 7577–7582 (2011). 21502501 10.1073/pnas.1103000108 PMC3088627

[30] Exley, R., McIntosh, J. M., Marks, M. J., Maskos, U. & Cragg, S. J. Striatal α5 nicotinic receptor subunit regulates dopamine transmission in dorsal striatum. *J. Neurosci.* **32**, 2352–2356 (2012). 22396410 10.1523/JNEUROSCI.4985-11.2012 PMC3742968

[31] Collins, A. L., Aitken, T. J., Greenfield, V. Y., Ostlund, S. B. & Wassum, K. M. Nucleus accumbens acetylcholine receptors modulate dopamine and motivation. *Neuropsychopharmacology* **41**, 2830–2838 (2016). 27240658 10.1038/npp.2016.81 PMC5061892

[32] Sun, F. et al. Next-generation GRAB sensors for monitoring dopaminergic activity in vivo. *Nat. Methods* **17**, 1156–1166 (2020). 33087905 10.1038/s41592-020-00981-9 PMC7648260

[33] Cunningham, C. L., Gremel, C. M. & Groblewski, P. A. Drug-induced conditioned place preference and aversion in mice. *Nat. Protoc.* **1**, 1662–1670 (2006). 17487149 10.1038/nprot.2006.279

[34] Collins, A. L. et al. Nucleus accumbens cholinergic interneurons oppose cue-motivated behavior. *Biol. Psychiat.* **86**, 388–396 (2019). 30955842 10.1016/j.biopsych.2019.02.014 PMC7003647

[35] Aosaki, T., Graybiel, A. M. & Kimura, M. Effect of the nigrostriatal dopamine system on acquired neural responses in the striatum of behaving monkeys. *Science* **265**, 412–415 (1994). 8023166 10.1126/science.8023166

[36] Kramer, P. F. et al. Synaptic-like axo-axonal transmission from striatal cholinergic interneurons onto dopaminergic fibers. *Neuron*10.1016/j.neuron.2022.07.011 (2022). 10.1016/j.neuron.2022.07.011 PMC9509469 35931070

[37] Zhang, H. & Sulzer, D. Frequency-dependent modulation of dopamine release by nicotine. *Nat. Neurosci.* **7**, 581–582 (2004). 15146187 10.1038/nn1243

[38] Kosillo, P., Zhang, Y. F., Threlfell, S. & Cragg, S. J. Cortical control of striatal dopamine transmission via striatal cholinergic interneurons. *Cereb. Cortex* **26**, 4160–4169 (2016). 27566978 10.1093/cercor/bhw252 PMC5066833

[39] Cragg, S. J. Meaningful silences: how dopamine listens to the ACh pause. *Trends Neurosci.* **29**, 125–131 (2006). 16443285 10.1016/j.tins.2006.01.003

[40] Schultz, W. Getting formal with dopamine and reward. *Neuron* **36**, 241–263 (2002). 12383780 10.1016/s0896-6273(02)00967-4

[41] Howe, M. W. & Dombeck, D. A. Rapid signalling in distinct dopaminergic axons during locomotion and reward. *Nature* **535**, 505–510 (2016). 27398617 10.1038/nature18942 PMC4970879

[42] Willuhn, I., Burgeno, L. M., Groblewski, P. A. & Phillips, P. E. Excessive cocaine use results from decreased phasic dopamine signaling in the striatum. *Nat. Neurosci.* **17**, 704–709 (2014). 24705184 10.1038/nn.3694 PMC4714770

[43] Cragg, S. J., Hille, C. J. & Greenfield, S. A. Functional domains in dorsal striatum of the nonhuman primate are defined by the dynamic behavior of dopamine. *J. Neurosci.* **22**, 5705–5712 (2002). 12097522 10.1523/JNEUROSCI.22-13-05705.2002 PMC6758186

[44] Willuhn, I., Burgeno, L. M., Everitt, B. J. & Phillips, P. E. Hierarchical recruitment of phasic dopamine signaling in the striatum during the progression of cocaine use. *Proc. Natl Acad. Sci. USA* **109**, 20703–20708 (2012). 23184975 10.1073/pnas.1213460109 PMC3528544

[45] Hamid, A. A., Frank, M. J. & Moore, C. I. Wave-like dopamine dynamics as a mechanism for spatiotemporal credit assignment. *Cell*10.1016/j.cell.2021.03.046 (2019). 10.1016/j.cell.2021.03.046 PMC8122079 33861952

[46] Zhang, Y.-F., Fisher, S. D., Oswald, M., Wickens, J. R. & Reynolds, J. N. J. Coincidence of cholinergic pauses, dopaminergic activation and depolarization drives synaptic plasticity in the striatum. *Nat. Commun.*10.1038/s41467-022-28950-0 (2019). 10.1038/s41467-022-28950-0 PMC8917208 35277506

[47] Zhang, Y.-F. et al. An axonal brake on striatal dopamine output by cholinergic interneurons. Zenodo 10.5281/zenodo.13898624 (2025). 10.1038/s41593-025-01906-5 PMC11976267 40082616

[48] Zhang, Y.-F. & Cragg, S. J. An axonal brake on striatal dopamine output by cholinergic interneurons. *protocols.io*10.17504/protocols.io.kqdg3q77ev25/v1 (2025). 10.1038/s41593-025-01906-5 PMC11976267 40082616

[49] Zhang, Y.-F. craggASAP/Axonal_Brake: Custom MATLAB scripts related to Zhang (2025) “An axonal brake on striatal dopamine output by cholinergic interneurons”. *Zenodo*10.5281/zenodo.14622173 (2025). 10.1038/s41593-025-01906-5 PMC11976267 40082616

