# Supplementary material for: An axonal brake on striatal dopamine output by cholinergic interneurons
Source: Nat Neurosci. 2025 Mar 13;28(4):783–94. doi: 10.1038/s41593-025-01906-5 (PMC11976267; doi:10.1038/s41593-025-01906-5)
Supplement: Supplementary file 1 — Reporting Summary [file 41593_2025_1906_MOESM1_ESM.pdf]

nature portfolio

Corresponding author(s): Yanfeng Zhang, Stephanie Cragg

Last updated by author(s): Feb 26, 2025

Reporting Summary

Nature Portfolio wishes to improve the reproducibility of the work that we publish. This form provides structure for consistency and transparency in reporting. For further information on Nature Portfolio policies, see our Editorial Policies and the Editorial Policy Checklist.

Statistics

For all statistical analyses, confirm that the following items are present in the figure legend, table legend, main text, or Methods section.

n/a	Confirmed	
☐	☒	The exact sample size (*n*) for each experimental group/condition, given as a discrete number and unit of measurement
☐	☒	A statement on whether measurements were taken from distinct samples or whether the same sample was measured repeatedly
☐	☒	The statistical test(s) used AND whether they are one- or two-sided<br>*Only common tests should be described solely by name; describe more complex techniques in the Methods section.*
☒	☐	A description of all covariates tested
☐	☒	A description of any assumptions or corrections, such as tests of normality and adjustment for multiple comparisons
☐	☒	A full description of the statistical parameters including central tendency (e.g. means) or other basic estimates (e.g. regression coefficient) AND variation (e.g. standard deviation) or associated estimates of uncertainty (e.g. confidence intervals)
☐	☒	For null hypothesis testing, the test statistic (e.g. *F*, *t*, *r*) with confidence intervals, effect sizes, degrees of freedom and *P* value noted<br>*Give P values as exact values whenever suitable.*
☒	☐	For Bayesian analysis, information on the choice of priors and Markov chain Monte Carlo settings
☒	☐	For hierarchical and complex designs, identification of the appropriate level for tests and full reporting of outcomes
☒	☐	Estimates of effect sizes (e.g. Cohen's *d*, Pearson's *r*), indicating how they were calculated

Our web collection on statistics for biologists contains articles on many of the points above.

Software and code

Policy information about availability of computer code

Data collection	PClamp v10.5; Fiji v1.5; Bonsi; Smart V3.0; Micro-Manager v1.4; Igor Pro v6; Fluoview; ZEN black v2.3
Data analysis	PClamp v10.5; Fiji v1.5; Smart v3.0; MATLAB vR2019b; GraphPad Prism v6

For manuscripts utilizing custom algorithms or software that are central to the research but not yet described in published literature, software must be made available to editors and reviewers. We strongly encourage code deposition in a community repository (e.g. GitHub). See the Nature Portfolio guidelines for submitting code & software for further information.

Data

Policy information about availability of data

All manuscripts must include a data availability statement. This statement should provide the following information, where applicable:

- Accession codes, unique identifiers, or web links for publicly available datasets
- A description of any restrictions on data availability
- For clinical datasets or third party data, please ensure that the statement adheres to our policy

Source data are provided with this paper and also available on Zenodo (http://doi.org/10.5281/zenodo.13898624).

Human research participants

Policy information about studies involving human research participants and Sex and Gender in Research.

Reporting on sex and gender	N/A
Population characteristics	N/A
Recruitment	N/A
Ethics oversight	N/A

Note that full information on the approval of the study protocol must also be provided in the manuscript.

Field-specific reporting

Please select the one below that is the best fit for your research. If you are not sure, read the appropriate sections before making your selection.

☒ Life sciences ☐ Behavioural & social sciences ☐ Ecological, evolutionary & environmental sciences

For a reference copy of the document with all sections, see nature.com/documents/nr-reporting-summary-flat.pdf

Life sciences study design

All studies must disclose on these points even when the disclosure is negative.

Sample size	Sample sizes in this study are listed in the main text, and corresponding figure legends. Sample sizes employed were based on previous FCV, imaging and behavioural experiments (Condon et al., 2019). Sample size were not pre-determined by formal power calculations.
Data exclusions	All the data collected for the study are included.
Replication	All attempts (> 3 times) at replication were successful.
Randomization	The animals are randomly grouped to ensure the randomization of the experiments.
Blinding	The Investigators were not blinded to allocation during experiments and outcome assessment as knowledge of experimental paradigm was important to ensure conduct of appropriate experiment.

Reporting for specific materials, systems and methods

We require information from authors about some types of materials, experimental systems and methods used in many studies. Here, indicate whether each material, system or method listed is relevant to your study. If you are not sure if a list item applies to your research, read the appropriate section before selecting a response.

Materials & experimental systems

n/a	Involved in the study	
☐	☒	Antibodies
☒	☐	Eukaryotic cell lines
☒	☐	Palaeontology and archaeology
☐	☒	Animals and other organisms
☒	☐	Clinical data
☒	☐	Dual use research of concern

Methods

n/a	Involved in the study	
☒	☐	ChIP-seq
☒	☐	Flow cytometry
☒	☐	MRI-based neuroimaging

Antibodies

Antibodies used	Primary antibodies: Goat anti-Choline Acetyltransferase (ChAT) (Sigma-Aldrich; Cat# SAB2500233; RRID:AB_10603703), Goat anti-Choline Acetyltransferase (ChAT) (Sigma-Aldrich; Cat# AB144P; RRID:AB_2079751), Rabbit anti-Tyrosine Hydroxylase (TH) (Sigma-Aldrich; Cat# T8700; RRID:AB_1080430). Secondary antibodies: Alexa Fluor 568 Donkey anti-Goat (Thermo Fisher Scientific Cat# A-11057, RRID:AB_2534104), AMCA-conjugated Donkey anti-Goat (Jackson ImmunoResearch Labs; Cat# 705-155-147; RRID:AB_2340409), Goat anti-Rabbit DyLight® 594 (Abcam; Cat# ab96885; RRID:AB_10680092), Goat Anti-Rabbit CoraLite488 conjugated Affinipure (Proteintech; Cat# SA00013-2; RRID:AB_2797132).

Validation	Goat anti-Choline Acetyltransferase (ChAT) primary antibody (Sigma-Aldrich, Cat# AB144P; RRID:AB_2079751) was validated by using Western Blotting (https://www.merckmillipore.com/GB/en/product/Anti-Choline-Acetyltransferase-Antibody,MM_NF-AB144P#documentation); Rabbit anti-Tyrosine Hydroxylase (TH) primary antibody (Sigma-Aldrich; Cat# T8700; RRID:AB_1080430) was validated by using western blotting (https://www.sigmaaldrich.com/GB/en/product/sigma/t8700srsltid=AfmBOoqJRUicR9W0i3TOsYeZOXfQaMB6oAXWuULkvtq1hzMp5mBWCXZo).

Animals and other research organisms

Policy information about studies involving animals; ARRIVE guidelines recommended for reporting animal research, and Sex and Gender in Research

Laboratory animals	Mice used in ex vivo experiments and in vivo DA recordings were adult wild-type C57BL/6J mice (Charles River, UK; RRID:IMSR_JAX:000664) (21-40 days), heterozygous ChAT-Cre:Ai32 (6-16 weeks), heterozygous DAT-Cre:Ai95D (4-7 weeks), heterozygous DAT-Cre:ChAT-Cre mice (8-12 weeks), or heterozygous DAT-IRES-Cre (B6.SJL-Slc6a3tm1.1(cre)Bkmn/J, JAX stock number 006660) (8-16 weeks) injected with viral vectors. Heterozygote ChAT-Cre:Ai32 mice were generated from ChAT-Cre+/+ mice (B6;129S6-Chattm2(cre)Lowl/J; JAX; Cat# 006410; RRID:IMSR_JAX:006410) crossed with Ai32+/+ mice (B6;129S-Gt(ROSA)26Sortm32(CAG-COP4*H134R/EYFP)Hze/J; JAX; Cat# 012569; RRID:IMSR_JAX:012569). Heterozygote DAT-Cre:Ai95D mice were generated from DAT-IRES-Cre+/- mice crossed with Ai95D+/+ mice (B6:129S-Gt(ROSA)26Sortm95.1(CAG-GCaMP6f)Hze/J; JAX; Cat# 024105; RRID:IMSR_JAX:024105). Male C57BL/6N mice (Charles River, Beijing, China; RRID:MGI:2159965) (42-50 days) were used for behavioral experiments. Animals were maintained at 20-24 deg C, humidity 40-60%.
Wild animals	No wild animals were used in this study.
Reporting on sex	Mice of both sexes were used except in some components of the study where male only mice are indicated (behavioural components). Data were not disaggregated for sex.
Field-collected samples	No field-collected samples were used in the study.
Ethics oversight	The procedures for ex vivo recordings and anaesthetised in vivo DA recordings were performed in accordance with Animals (Scientific Procedures) Act 1986 (Amended 2012) with ethical approval from the University of Oxford, and under authority of a Project Licence granted by the UK Home Office. Behavioural experiments were performed using protocols approved by the Animal Care & Use Committees at the Chinese Institute for Brain Research (#CIBR-IACUC-007) and were performed in accordance with the guidelines established by US National Institutes of Health.

Note that full information on the approval of the study protocol must also be provided in the manuscript.
